# Care at a distance: Understanding lived experiences of people with MSK disorders receiving non-pharmacological interventions delivered through synchronous telehealth: A systematic rapid review

**DOI:** 10.1177/20552076241236573

**Published:** 2024-03-15

**Authors:** Melissa Atkinson-Graham, Ginny Brunton, Carol Cancelliere, Melissa Corso, Annemarie de Zoete, Sidney M Rubinstein, Kent Murnaghan, Silvano Mior

**Affiliations:** 1 Institute for Disability and Rehabilitation Research, 85458 Ontario Tech University, Oshawa, ON, Canada; 2 Division of Research and Innovation, 7948 Canadian Memorial Chiropractic College, Toronto ON, Canada; 3 Faculty of Health Sciences, and Institute for Disability and Rehabilitation Research, 85458 Ontario Tech University, Oshawa, ON, Canada; 4 Department of Health Sciences, 1190 Vrije Universiteit Amsterdam, Amsterdam, The Netherlands; 5 Library Sciences, 7948 Canadian Memorial Chiropractic College, Toronto ON, Canada

**Keywords:** Telehealth, musculoskeletal, rehabilitation, qualitative, systematic reviews, patient experience

## Abstract

**Background:**

Little is known about lived experience of synchronous telehealth in patients with musculoskeletal (MSK) disorders.

**Objective:**

We conducted a rapid systematic review to answer: (1) what are the lived experiences and/or perspectives of people with MSK disorders receiving non-pharmacological interventions delivered through synchronous telehealth; and (2) what clinical implications can be inferred from qualitative studies focusing on lived experiences for how telehealth is delivered in the management of MSK disorders?

**Data sources:**

A comprehensive search of MEDLINE, CINAHL, PsycINFO, ProQuest, and Google Scholar from June 2010 to July 2023. Eligible qualitative and mixed methods studies capturing lived experiences of adults with MSK disorders receiving non-pharmacological interventions via synchronous telehealth were included.

**Study methods:**

Systematic rapid review conducted according to WHO guidelines. Titles and abstracts screened by reviewers independently, eligible studies critically appraised, and data was extracted. Themes summarized using the Consolidated Framework for Implementation Research (CFIR). GRADE-CERQual (Confidence in the Evidence from Reviews of Qualitative research) used to assess confidence in synthesis findings.

**Results:**

We identified 9782 references, screened 8029, and critically appraised 22, and included 17 studies. There is evidence to suggest that the experience of telehealth prior to and during the pandemic was shaped by (1) patient perception of telehealth, (2) existing relationships with practitioners, (3) availability and accessibility of telehealth technologies, and (4) perceptions about the importance of the role of the physical exam in assessing and treating MSK disorders.

**Conclusion:**

The five identified implications could be used to inform future research, policy, and strategy development.

## Introduction

In the wake of the COVID-19 pandemic, we saw a dramatic uptake in the use of digital health technologies, including telehealth. The global pandemic instantiated a significant shift toward the use of telehealth in the non-pharmacological management of musculoskeletal (MSK) conditions.^1–4^ Over the past decade, evidence-based guidelines have advocated for the role of non-pharmacological interventions for the management of MSK conditions.^2,5–8^ We understand these interventions include but are not limited to education and reassurance, exercise-based rehabilitation, manual therapy, physical therapies, cognitive behavioral therapy, and other conservative interventions focused on reducing pain and improving function and quality of life. Previous reviews on the role of telehealth in the non-pharmacological management of MSK conditions have astutely noted that many of these guidelines recommended in-person and clinic-based interventions.^9^ To this end, the use of telehealth in MSK care has raised several concerns regarding the adaptability of such hands-on and in-person practices to this emerging paradigm of remote care.^10–12^ Nonetheless, research efforts during the pandemic have demonstrated encouraging results regarding the efficacy and safety of telehealth for the management of MSK disorders, which have been summarized in recent reviews.^9,13^ Despite these studies, less is known about the lived experience of synchronous telehealth from the perspective of patients with MSK disorders and how these experiences shifted during the pandemic.

Against growing evidence supporting the use of telehealth in MSK care,^2,4,9,14–17^ further understanding of the emotional, psychosocial, political, economic, and cultural experiences of patients is required. To date, much of the scholarship on the use of telehealth in MSK care has focused on barriers and enablers encountered by users of telehealth,^12,18^ questions of patient satisfaction,^19^ and the experiences of healthcare providers using this approach to care.^3,20^ For the purposes of our review, we are concerned with the nature of the experience of using synchronous telehealth interventions from the perspectives of patients, and the broader experiential context in which telehealth as method of MSK care delivery is situated. This knowledge is needed in order to meaningfully implement telehealth as a person-centered practice thereby expanding the reach of MSK care, rather than being a tool that substitutes for in-person care in emergency and extenuating circumstances.^15^ To this end, our review investigates two intertwined questions:

1. what are the lived experiences and/or perspectives of people with MSK disorders receiving non-pharmacological interventions delivered through synchronous telehealth? and
2. what clinical implications can be inferred from qualitative studies that focus on lived experiences for how telehealth is delivered in the management of MSK disorders?

## Methods

### Protocol and registration

Our systematic rapid review was conducted in accordance with WHO guidelines.^21^ It builds on a recently conducted rapid quantitative evidence synthesis^9^ which sought to determine whether non-pharmacological interventions delivered through synchronous telehealth are as effective and safe as clinic-based in-person interventions for improving outcomes, such as pain, functioning, self-reported recovery for the management of patients with MSK conditions. Our original protocol was registered on the Open Science Framework (OSF) on 8 August 2021 (osf.io/vm7by) and updated on 12 June 2022. An updated literature search was performed on July 2023. Due to unforeseen circumstances and challenges related to the pandemic, our timeline was extended. Nevertheless, our rapid review approach is consistent with Cochrane guidance on rapid review methods insofar as our approach streamlined the knowledge synthesis and produced evidence in a “resource-efficient manner.”^22^

### Ethics statement

No research ethics review was sought for this review as our analysis of patient experiences utilizes published and publicly reported qualitative literature.

### Information sources

A health science librarian developed a comprehensive search strategy (see Appendix A). Three concept groups were used: (1) MSK disorders, (2) telehealth, and (3) qualitative research. Search terms included subject headings specific to each database (e.g. MeSH in MEDLINE) and free text words relevant to our objectives and eligible study designs. We systematically searched MEDLINE (Ovid), CINAHL (EBSCO), and PsycINFO (Ovid), as well as ProQuest Dissertations & Theses Global and Google Scholar from June 2010 to June 2023. The date range of our search reflects technological and digital advancements impacting telehealth delivery in the past decade, most notably the emergence of smartphones and tablets to global markets in 2010. Prior to the COVID-19 pandemic, telehealth was primarily deployed in the context of emergency situations, including its use in military crisis managed by The North Atlantic Treaty Alliance (NATO) in 2000, and later during several environmental disasters in the United States and Australia in 2017 and 2019.^23^ This date range thus reflects important technological and historical events shaping telehealth.

### Eligibility criteria

Eligibility criteria was organized using the Sample, Phenomenon of Interest, Design, Evaluation, Research type (SPIDER) Tool for Qualitative Evidence Synthesis.^24,25^

#### Sample

We included studies of adult populations with MSK disorders receiving non-pharmacological interventions offered via synchronous telehealth. We define MSK disorders as conditions that affect the locomotor system including injuries or disorders of the muscles, nerves, tendons, joints, cartilages, and supporting structures.^26,27^ We excluded conditions such as stroke, post-operative rehabilitation, fracture, or inflammatory arthropathies which require specific rehabilitation equipment, programs, or co-management with medical teams.

#### Phenomenon of interest

We investigated the lived experience of patients receiving non-pharmacological interventions offered via synchronous telehealth to treat MSK disorders. We define telehealth as the delivery of healthcare services, where patients and providers are separated by distance and exchange information regarding the diagnosis and treatment of diseases and injuries, research and evaluation, and education. Synchronous telehealth involves the delivery of healthcare services in real-time (e.g. through videoconferencing or telephone).^28^ Asynchronous telehealth interventions (e.g. apps, text-based services, web portals, virtual reality, or pre-recorded video demonstrations) were excluded.

#### Design

We included qualitative studies of a range of qualitative methodologies. Studies were required to be experientially descriptive, and include quotes or illustrative passages describing the emotional, psychological, social, historical, political, economic dimensions of telehealth.

#### Evaluation

Our review focused on understanding the experience of telehealth from the perspective of patients. We defined lived experience as both the quotidian moments, decisions, and actions that shape a person's everyday life, and the forms of meaning that a person makes or collects about these experiences. We acknowledge that lived experience is shaped by formations of race, ethnicity, class, gender, and sex and their intersections; and that lived experience has emotional, psychological, sociocultural, historical, political, economic dimensions that are both tangible and intangible, articulable and inarticulable.

#### Research type

We included empirical studies published in English, in peer-reviewed journals representative of a range of qualitative methodologies. As well as studies using semi-structured interviews with individuals and groups conducted in-person, over the phone, and virtually. Mixed method studies were considered when the qualitative components could be extracted for appraisal and appropriately evaluated for adherence to quality criteria of its tradition following Mixed Methods Appraisal Tool (MMAT) guidance.^29^ We also searched reference lists of all eligible articles for additional relevant studies. A grey literature search which included Google Scholar was conducted.

### Study selection

We utilized EPPI-Reviewer software to conduct all screening and critical appraisal efforts.^30^ In keeping with rapid review methodology,^21,22^ the lead author, a chiropractor in training with a doctorate in social anthropology, screened all the records and full texts. A second experienced clinician and researcher screened a random sample of 10% of the records to ensure that the selection was conducted appropriately.

Screening was based on our inclusion criteria, and citations were labeled relevant or possibly relevant. Full text screening was conducted by reviewing full texts of all relevant and possibly relevant articles. Disagreements regarding study eligibility were resolved through discussion. Unresolved disagreements were discussed, and consensus reached with a third author.

### Critical appraisal

Critical appraisal was conducted on eligible studies by two experienced clinicians and researchers using the Critical Appraisals Skills Program (CASP) tool^31^ or the MMAT tool^29^ (Table 1a and b). No relevant gray literature was identified for critical appraisal. Appraisal rating disagreements were resolved through team discussions.

**Table 1.** Critical appraisal results.

	**Table 1.a—CASP**									
	Screening question		Detailed questions							
	1. Was there a clear statement of the aims of the research?	2. Is a qualitative methodology appropriate?	3. Was the research design appropriate to address the aims of the research?	4. Was the recruitment strategy appropriate to the aims of the research?	5. Was the data collected in a way that addressed the research issue?	6. Has the relationship between researcher and participants been adequately considered?	7. Have ethical issues been taken into consideration?	8. Was the data analysis sufficiently rigorous?	9. Is there a clear statement of findings?	10. How valuable is the research?
Bell et al.^32^	Yes	Yes	No	No	Yes	No	Yes	Can’t tell	Yes	Somewhat valuable
Booth et al.^33^	Yes	Yes	Yes	Yes	Yes	Yes	Yes	Yes	Yes	Valuable
Ernstzen et al.^34^	Yes	Yes	Yes	Yes	Yes	Yes	Yes	Yes	Yes	Valuable
Ezzat et al.^35^	Yes	Yes	Yes	Yes	Yes	Can’t tell	Yes	Yes	Yes	Valuable
Fraser et al.^36^	Yes	Yes	Yes	Yes	Yes	Yes	Yes	Yes	Yes	Valuable
Gilbert et al.^37^	Yes	Yes	Yes	Yes	Yes	Yes	Yes	Yes	Yes	Valuable
Gilbert et al.^38^	Yes	Yes	Yes	Yes	Yes	Yes	Yes	Yes	Yes	Valuable
Hasani et al.^39^	Yes	Yes	Yes	Yes	Yes	Yes	Yes	Yes	Yes	Valuable
Hinman et al.^40^	Yes	Yes	Yes	Yes	Yes	Yes	Yes	Yes	Yes	Valuable
Hinman et al.^41^	Yes	Yes	Yes	Yes	Yes	Yes	Yes	Can’t tell	Yes	Somewhat valuable
Pearson et al.^42^	Yes	Yes	Yes	Yes	Yes	No	Yes	Yes	Yes	Somewhat valuable
Ryan et al.^43^	Yes	Yes	Yes	Yes	Yes	Yes	Yes	Yes	Yes	Valuable

	**Table 1.b—MMAT**											
	**Screening questions**		**Qualitative**					**Mixed methods**				
	**S1. Are there clear research questions?**	**S2. Do the collected data allow to address the research questions?**	**1.1. Is the qualitative approach appropriate to answer the research question?**	**1.2. Are the qualitative data collection methods adequate to address the research question?**	**1.3. Are the findings adequately derived from the data?**	**1.4. Is the interpretation of results sufficiently substantiated by data?**	**1.5. Is there coherence between qualitative data sources, collection, analysis and interpretation?**	**5.1. Is there an adequate rationale for using a mixed methods design to address the research question?**	**5.2. Are the different components of the study effectively integrated to answer the research question?**	**5.3. Are the outputs of the integration of qualitative and quantitative components adequately interpreted?**	**5.4. Are divergences and inconsistencies between quantitative and qualitative results adequately addressed?**	**5.5. Do the different components of the study adhere to the quality criteria of each tradition of the methods involved?**
Barton et al.^44^	Yes	Yes	Yes	Yes	Yes	Can’t tell	No	Yes	No	No	No	No
Cottrell et al.^45^	Yes	Yes	Yes	Yes	Yes	Yes	Yes	Can’t tell	No	Can’t tell	No	Yes
Dharmasri et al.^46^	Yes	Yes	Yes	No	Yes	Can’t tell	Yes	Yes	Yes	Yes	Yes	No
Thesen et al.^47^	Yes	Yes	Yes	Yes	Yes	Yes	Yes	Yes	Yes	Yes	Yes	Yes
Skolasky et al.^48^	Yes	Yes	Yes	Yes	Yes	Yes	Can’t tell	Yes	Yes	Yes	Can’t tell	Can’t tell

### Data extraction

Data extraction was conducted by the lead author. We utilized guidance from the National Institute for Health and Care Excellence (NICE) to construct our evidence table^49^ capturing data on study research questions, participant characteristics, setting, sample size, and identified themes (Table 2). Themes and subthemes discussed in each of the included articles were numbered for organizational clarity. The extracted data was reviewed and verified by the research team.

**Table 2.** Qualitative evidence summary table^a^.

Study details	Research parameters	Population and sample selection	Outcomes and methods of analysis results	Notes by reviewer
Barton et al.^44^ **Quality score: +**	Research question: To explore the experiences and attitudes of patients receiving physiotherapy telehealth services for musculoskeletal pain conditions during the COVID-19 pandemic. Theoretical approach: Non stated Data collection: Sequential mixed-methods explanatory design using individual semi-structured interviews over Zoom.	Country of study: Australia Population selection: Patients recruited from > 200 private physiotherapy clinics across Australia. Sample selection: Purposive sampling Recruitment approach: Flyer advertisements in clinics. Patients invited to complete a quantitative survey and if consented to follow-up contact were invited to a qualitative interview. Total participants recruited: 186 people consented. 172 completed the survey. Mean age of survey cohort 49 yrs. 62% women, 38% men. 19 participants (60% women) with a mean age of 53 (SD = 17) years were interviewed prior to reaching data saturation (part 2). Inclusion criteria: Agreed and consented to be contacted via email. Otherwise not stated. Exclusion criteria: Not stated	Method and process of analysis: Thematic Key themes: 1. ‘Telehealth had value, but generally perceived as inferior to in-person care’ 1.1 Valuable if in-person care is not an option 1.2 Surprise at value, after initial hesitation 1.3 Part of hybrid model in certain situations or conditions 1.4 Cost should be the same or less 1.5 Existing physiotherapist-patient relationships was considered valuable to facilitate telehealth) 2. ‘Challenges related to assessment, diagnosis, ‘hands on’ treatment, observation, communication, and technology’ 2.1 Accurate assessment and diagnosis 2.1 lack of hands-on treatment 2.3 Difficulty with observation 2.4 Understanding and communicating 2.5 Perceived as less personal typically but not always 2.6 Technology concerns) 3. ‘Advantages to access safe, expert, and convenient care’ 3.1 Facilitates access to the right expertise 3.2 Convenience of not having to travel, 3.3 No risk of contracting COVID 3.4 Exercises in home environment 4. ‘Importance of supportive technology, including video and supplementary resources’ 4.1 Video calls important 4.2 Supplemental resources	Limitations identified by author: Patients recruited from private physiotherapy clinics and findings may not reflect publicly funded healthcare settings, or international context. Participants had necessary resources available for telehealth (financial, hardware, internet access), and a minority of participants reported comorbidities. Study took place during COVID-19 lock downs in 2020, which may impact the perceived value of telehealth. Interview sample may have been biased by positive telehealth experiences. Source of funding: None stated Evidence gaps and/or recommendations for future research: To explore the value of telehealth as part of a hybrid model, and how to enhance therapeutic alliance and communication during the delivery of telehealth services.
Booth et al.^33^ **Quality score + **	Research question: The primary objective was to identify factors that lead to the virtual pain management programme (vPMP) being acceptable or not acceptable. Secondary objectives were to identify factors that might lead to a patient being more suited to a remotely delivered pain management programme, gather considerations for future programmes and to make recommendations for clinical practice and future research. Theoretical approach: Theoretical Framework of Acceptability (TFA) Data collection: Microsoft Teams based remote focus group of 3–5 patients per group.	Country of study: United Kingdom Population selection: Participants who had completed a vPMP delivered at a tertiary musculoskeletal hospital in North London. Sample selection: Variation across vPMP cohorts Recruitment approach: Participants who had completed vPMP between 2020–2021 were contacted by phone and provided information about the evaluation Total participants recruited: 13. Mean age 42 years (range 19–72 years). 10 female, 3 male. Inclusion criteria: Completed vPMP program between 2020–2021 Exclusion criteria: Participated in vPMP pilot cohort, difficulty with telephone communication, in ability to participate in a recorded Microsoft Teams based remote focus group.	Method of analysis: Abductive Key themes: 1. The vPMP is entirely acceptable 1.1 Learning to self-manage persistent pain whilst at home 1.2 Receiving a high quality of care from home 1.3 Enhancing the potential of rehabilitation using technology 1.4 Enabling pain management programme attendance 1.5 Overcoming social distancing requirements of COVID-19 through technology 1.6 Virtual peer support 2. The vPMP is not acceptable at all 2.1 Inappropriate home environment for virtual therapy 2.2 Communication challenges with virtually delivered care 2.3 Technological issues 2.4 Concerns about the quality of remotely delivered care	Limitations identified by author: Focus group discussions were not entirely transcribed verbatim; multiple authors listened to recordings and noted key insights. Participants in vPMP and study have confidence in videoconferencing and this could have biased their view on vPMP acceptability. Sources of funding: None stated Evidence gaps and/or recommendations for future research: Examine acceptability of remote programmes from clinician perspective; compare the acceptability of in-person and remote pain management programmes, evaluate the reasons for patients not opting for a remote programme in a wider patient group and investigate the effectiveness of in-person, virtual and hybrid pain management programmes.
Bell et al.^32^ **Quality score: +**	Research question: This study aimed to better understand how this rapid implementation of telemedicine [during the COVID-19 pandemic] impacted health care delivery in the rural state of Maine. Theoretical approach: None stated Data collection: Structured interviews; limited choice responses, open-ended questions	Country of study: United States Population selection: Patients of a small, private practice in rural Maine Sample selection: Purposive Sampling Recruitment approach: Email invitation sent to patients who had participated in a video telemedicine appointment. Total participants recruited: 14 patients. Average age 65.6. 57% female, 43% male. 99% white. 35% insured by Medicare or Medicaid Sample Selection: Purposive sample. Inclusion criteria: Patients who participated in a video telemedicine appointment between April 1–31 2020. Exclusion criteria: Not stated	Method and process of analysis: Thematic Key themes: 1. Convenience 2. Perceived efficacy 3. Alterations in the ritual of medicine 4. Emotional connection 5. Feasibility 6. Future directions	Limitations identified by author: Homogeneous sample population across age, race, insurance coverage. Representative of older, white population. Source of funding: Grant U54 GM115516 from the National Institutes of Health for the Northern New England Clinical and Translational Research network. Evidence gaps and/or recommendations for future research: Continued research on the benefits and challenges of telemedicine for patients and providers.
Cottrell et al.^45^ **Quality score: +**	Research question: To (a) explore stakeholder perspectives towards the delivery of Allied Health Professions (AHP) healthcare via telehealth during COVID-19 and (b) examine factors influencing implementation to inform the enhancement and sustainability of AHP telehealth services. Theoretical approach: None stated Data collection: Open ended survey question, semi-structured telephone interviews.	Country of study: Australia Population selection: Patients from Royal Brisbane and Women's Hospital (RBWH) in Queensland, Australia, who had received telehealth. Sample selection: Convenience sampling Recruitment approach: Patients who participated in the survey and consented to contact were contacted directly to participate in the interviews. Total participants recruited: 109 patients. 58% female, 51% male. 56% between 40–59 years, 33% between 16–39, 19% 70 + years (average age 36). Inclusion criteria: > 16 years, received outpatient care via telehealth from a single department in RBWH during the mandated COVID-19 restrictions (March-June 2020 inclusive). Exclusion criteria: Not stated	Method and process of analysis: Thematic and template analysis Key themes: 1. Experience of telehealth was overall positive 1.1 Grateful for the opportunity to continue access to care during COVID-19 restrictions 1.2 Telehealth service provided convenience, reduced waiting and travel time and reduced cost 1.3 Avoiding hospital when immunocompromised reduced anxiety and supported feeling of safety during COVID restrictions 2. A blended model of telehealth and in-person consults is preferred 2.1 Preferred mode of delivery is dependent on appointment type or nature of tasks required for that appointment 2.2 Having the choice of a mix of in-person and telehealth appointments is preferred 3. Technology 3.1 Telehealth platform was easy to use 3.2 Inconsistent telehealth connection impacted experience 4. Support is required to engage in telehealth 4.1 More technology support is required 4.2 Patient support is required to engage with telehealth 4.3 Patient support is required to prepare for and engage in telehealth consults	Limitations identified by author: Survey response rate lower than expected, RBWH is a public quaternary facility and findings may not be generalizable to other facilities, professions, or privately funded models. Source of funding: Reports no financial support was received for research, authorship, or publishing of this article. Evidence gaps and/or recommendations for future research: How best to enhance and sustain AHP telehealth services.
Dharmasri et al.^46^ **Quality score: +**	Research Question: The objective of this secondary analysis of the STAART study (The Pain Coping Skills Training for African Americans with Osteoarthritis) was to report on participants’ responses to questions regarding their experiences and perceptions of the program, using a combination of quantitative and qualitative methods. Theoretical Approach: None stated Data Collection: Series of questions asked via telephone call.	Country of study: United States Population selection: 248 African Americans recruited from the University of North Carolina (UNC) healthcare system participating in the STAART study. Sample selection: None identified Recruitment approach: Invitation to self-referral into qualitative arm of a larger RCT Total participants recruited: 93 patients. Mean age 59.4. 50.5% female, 49.5% male Inclusion criteria: For STAART study: Self- reported diagnosis of knee or hip OA from a medical professional; Self-report of pain, aching, or stiffness in one or both knees or hips on most days of the week; Patient of UNC or DVAHCS. Exclusion criteria: None stated	Method and process of analysis: Thematic Key themes: 1. Improved Pain Coping 2. Mood and Emotional Benefits 3. Improved Physical Functioning 4. Intervention Delivery	Limitations identified by author: Small number of study participants. May not be generalizable because of study focus on African Americans with OA, many of whom are veterans. Study participants had relatively high levels of chronic illness. 25% of participants did not complete feedback questions. Some feedback questionnaires completed by intervention counselors. Source of funding: Patient-Centered Outcomes Research Institute (PCORI) Award (AD-1408–19519). Evidence gaps and/or recommendations for future research: Need to better understand sources of variability in patient satisfaction.
Ernstzen et al.^34^ **Quality score +**	Research question: Reporting on patients’ perspectives regarding participation in a telehealth intervention (ePEEP), and their perspectives on the acceptability and feasibility of ePEEP in the South African context. Theoretical approach: Qualitative, exploratory descriptive study design with a phenomenological approach Data collection: Individual semi- structured telephonic interviews.	Country of study: South Africa Population selection: Participants in ePEEP program, offered by interdisciplinary chronic pain management clinics Groote Schuur and Tygerberg Hospitals in Cape Town, South Africa. Sample selection: Purposive sampling Recruitment approach: Research team member contacted eligible ePEEP participants, obtained consent to be contacted by second team member. Second team member invited to study using text message and voice notes. Total participants recruited: 6 women. Mean age 43 years. Inclusion criteria: Completion of ePEEP; met ePEEP program inclusion criteria Exclusion criteria: ePEEP program exclusion criteria	Method of analysis: Inductive thematic content analysis Key Themes: 1. The journey of discovery 1.1 Departure point on journey 1.2 Meaningful learning 1.3 On reaching the journey destination 2. Building peer and therapeutic relationships 3. The online learning environment	Limitations identified by author: Gender bias; small sample size; structural and economic barriers to ePEEP participation. Sources of funding: None stated Evidence gaps and/or recommendations for future research: More research regarding clinical outcomes of ePEEP; role of gender in ePEEP; reasons for non-participation.
Ezzat et al.^35^ **Quality score +**	Research question: To understand patient perceived acceptability of participating in a telehealth delivered group- based education and exercise-therapy program for knee osteoarthritis Theoretical approach: interpretive description paradigm Data collection: Semi-structured, one-on-one interviews were conducted in-person or via videoconference.	Country of study: Australia Population selection: Participants with knee osteoarthritis were recruited from within an existing ongoing clinical trial comparing GLA:D® Australia delivered via telehealth or in-person. Sample selection: Sequential Recruitment approach: Contacted by email after completing the 8 week GLA:D® program, before 3 month follow-up assessment of the main trial. Total participants recruited: 19. Mean age 62 (range 49–72 years). 63% female, 37% male. Inclusion criteria: Trial inclusion criteria Exclusion criteria: None stated	Method of analysis: Thematic Key themes: 1. Participant perceptions of telehealth acceptability was highly influenced by exposure 1.1 Telehealth considered easy, convenient, and flexible by telehealth group, but inferior and misunderstood by in-person group 1.2 Telehealth was well supervised and quality feedback was provided 1.3 Technology hiccups could be worked out 2. Telehealth and in-person reported similar benefits of program 2.1 Reductions in fear of pain and joint damage resulting in improved confidence to engage in physical activity and exercise 2.2 Changed beliefs on importance and value of exercise 2.3 Self-reported improvements in pain and function	Limitations identified by author: Demographic information not computed; participants resided in urban areas and may not reflect rural experience; interviews conducted before COVID-19 pandemic. Sources of funding: None stated Evidence gaps and/or recommendations for future research: None stated
Fraser et al.^36^ **Quality score: **	Research question: The study aimed to explore participants’ treatment experiences [of telephone-delivered cognitive behavioral therapy] with a view to understanding their potential influences on intervention acceptability. Theoretical approach: None stated Data collection: Semi-structured in-depth telephone interviews.	Country of study: United Kingdom Population selection: Participants of the Maintain Musculoskeletal Health (MAmMOTH) study Sample selection: Purposive sampling Recruitment approach: Invitation sent to participants of MAmMOTH RCT who completed one treatment session and not withdrawn from treatment. Total participants recruited: 93 patients. Mean age 57.7. 50.5% female, 49.5% male Inclusion criteria: Participants of MAmMOTH RCT who completed one treatment session and not withdrawn from treatment. Exclusion criteria: Not stated	Method and process of analysis: Thematic Key themes: 1. Presenting issues and pain management 1.1 Nature of the pain experienced 1.2 Pain management strategies employed 1.3 Help received prior to participating in the trial 2. Expectations and reflections 2.1 Trial perceptions and motivations to participate 2.2 Initial expectations prior to therapy 2.3 Reflections on receiving the intervention 3. Intervention impact and acceptability 3.1 Post-intervention changes reported by participants	Limitations identified by author: Findings represent 11% of MAmMOTH trial cohort. Source of funding: Arthritis Research UK Grant no. 20748. Evidence gaps and/or recommendations for future research: Longitudinal studies needed to understand long term effects of tCBT intervention. Incorporate therapists’ views.
Gilbert et al.^37^ **Quality score: ++**	Research question: Qualitative exploration aimed to identify, characterize and explain factors that influence patient preferences for VCs (virtual consultation) in an orthopedic rehabilitation setting. Theoretical approach: Burden of Treatment Theory (BoT) Data collection: Phone or Skype based semi-structured interviews	Country of study: United Kingdom Population selection: Existing patients of an Occupational Therapy and Physiotherapy Department. Sample selection: Maximum variation Recruitment approach: Banner advertisements in the clinic, department wide emails to clinicians encouraging patient involvement. Total participants recruited: 22 patients. 55% women, 45% men. Average mean age of 46. Inclusion criteria: Patients, over the age of 18 years, attending host institutions for Physiotherapy or Occupational Therapy; have experience of orthopedic / musculoskeletal condition; are able to provide informed written consent to enter into the study; able to understand and speak English or a language covered by the host institution interpreter service. Exclusion criteria: Patients without the capacity to consent; suffering from disorders other than orthopedic as the primary cause (e.g. neurological or oncology disorders); those currently or previously treated by one of the researchers (AWG).	Method and process of analysis: Abduction, open coding Key themes: 1. The situation of care 1.1 Clinical status 1.2 Treatment requirements 1.3 Care pathway 2. Expectation of care 2.1 Desire for contact 2.2 Psychological status 2.3 Previous care 2.4 Perceived requirements 3. Demands of the patient 3.1 Care requirements 3.2 Social demands 3.3 Consequences of choice 4. Capacity to allocate resources to care 4.1 Financial 4.2 Infrastructure 4.3 Social capacity 4.4 Healthcare system	Limitations identified by author: Single center study. May not be generalizable. Study conducted prior to COVID-19 pandemic. Source of funding: National Institute for Health Research (NIHR) Evidence gaps and/or recommendations for future research: None stated
Gilbert et al.^38^ **Quality score: ++**	Research question: How are patient preferences for virtual consultations (VC) decided and organised following COVID-19? Theoretical approach: Normalization Process Theory (NPT) and Preference Theory Data collection: Individual interviews using phone or video call.	Country of study: United Kingdom Population selection: Existing patients of specialist orthopedic hospital in North London. Sample selection: pragmatic approach Recruitment approach: Hospital based clinicians asked to identify patients for participation; with consent email letter of invitation sent. Total participants recruited: 20 patients. Average age: 47 (age range: 22–74) 10 female, 10 male. Inclusion criteria: > 18 years old, attending the research site for physiotherapy or occupational therapy, experience of MSK condition, provide informed written consent; understand and speak English or language covered by the RNOH Interpreter service. Exclusion criteria: Patients without capability to consent, non-MSK disorders, patients previously treated by lead author.	Method of analysis: Abduction Key themes: 1. The context for the consultation 1.1 Normative expectations 1.2 Relational expectations 1.3 Congruence 1.4 Potential 2. The implementation process of VC 2.1 Coherence 2.2 Cognitive participation 2.3 Collective action 2.4 Reflexive monitoring 3.1 Results 3.1.1 The context for the consultation 3.1.2 The implementation process of VC 3.2 How Preferences for VC are decided 3.3 How preferences for VC are organized	Limitations identified by author: Constructions of preference may not be relevant post-COVID-19. Sources of funding: NIHR Research Trainees Coordinating Centre, Grant/Award Number: ICA-CDRF-2017- 03-025 Evidence gaps and/or recommendations for future research: Future research to understand preferences, research should be sensitive to patient preferences while acknowledging the preferred outcomes for patients, clinicians, organizations.
Hasani et al.^39^ **Quality score: +**	Research question: This qualitative study was nested within a pilot randomized trial and aimed to explore the experience of participants and physiotherapists with gym-based exercise interventions for Achilles tendinopathy with weekly telehealth monitoring (via videoconference). Theoretical approach: None stated. Data collection: Focus group using telehealth software	Country of study: Australia Population selection: Patients enrolled in the pilot RCT Sample selection: Purposive sampling Recruitment approach: Invited participants Total participants recruited: 8 participants. Average age 46. No sex/gender reported. Inclusion criteria: Patients who exhibited a range of self-reported primary outcome scores at final follow-up (12 weeks), and demonstrated acceptable and unacceptable adherence with the exercise intervention (acceptable adherence defined as achieving 66% or more of the number of sessions prescribed). Exclusion criteria: Not stated	Method and process of analysis: Inductive thematic analysis Key themes: 1. Acceptability of the telehealth sessions and gym-based exercise intervention 2. Enablers of adherence with telehealth monitoring 2.1 Usability of the software 2.2 Flexibility in arranging an appointment time 2.3 Therapeutic alliance 3. Barriers to adherence with telehealth monitoring 4. Enablers to adherence with the exercise interventions 4.1 Regular contact 4.2 The nature of the exercise 4.3 Accessibility to the gym 4.4 Self-management education 4.5 Ability to see progress 5. Barriers to adherence with the exercise interventions 5.1 Time 5.2 Exercise	Limitations identified by author: Sample size. Experiences may not be representative of the participants in primary trial. Selection bias. Participants who agreed may have had some positive feelings towards telehealth or gym rehabilitation to start with, despite many not having gym memberships. Source of funding: International Mechanical Diagnosis and Therapy Research Foundation (IMDTRF). Evidence gaps and/or recommendations for future research: None stated
Hinman et al.^40^ **Quality score: ++**	Research question: To explore how key stakeholders (physical therapists, telephone coaches, and patients) experienced, and made sense of, being involved in delivering or receiving an integrated physical therapy and telephone coaching intervention for osteoarthritis. Theoretical approach: Cross-sectional qualitative study drawing from symbolic interactionism. Data collection: Semi-structured individual phone interviews	Country of study: Australia Population selection: From large RCT Sample selection: Purposive sampling Recruitment approach: Approached 8 patients Total participants recruited: 6 patients. Mean age 62 50% female, 50% male. Inclusion criteria: Average knee pain > 4 on an 11-point numeric rating scale; American College of Rheumatology clinical classification criteria for knee OA (any 3 of the following criteria: 50 years or older, stiffness lasting less than 30 min, crepitus felt on passive or active movement of the knee, bony tenderness, bony enlargement, or no warmth to touch); classification of “sedentary” or “insufficient physical activity time” according to the Active Australia Survey. Exclusion criteria: Not stated	Method and process of analysis: Grounded Theory, thematic analysis; open coding followed categorization and theoretical analysis Key themes: 1. Genuine interest and collaboration 2. Information and accountability 3. Program structure 4. Roles and communication in teamwork	Limitations identified by author: Research conducted in Australia and unclear if findings are generalizable to other countries. Source of funding: National Health & Medical Research Council (Program Grant 631717). Evidence gaps and/or recommendations for future research: Investigate how best to integrate physical therapist-supervised exercise management with psychologically informed health coaching.
Hinman et al.^41^ **Quality score: ++**	Research question: The aim of this qualitative study was to explore the experience of patients and physical therapists using Skype as a service delivery model for physical therapist-prescribed exercise management of knee OA. Theoretical approach: Donabedian model for quality assessment in healthcare (based on grounded theory). Data collection: Telephone semi-structured interviews	Country of study: Australia Population selection: Recruited from RCT Sample selection: Purposive sampling Recruitment approach: Invited 3–6 months after completing RCT intervention Total participants recruited: 12. Mean age 62. 50% female. 50% male. Inclusion criteria: Patients with persistent knee pain associated with OA in the RCT Exclusion criteria: Not stated	Method and process of analysis: Thematic and constant comparative analytic Key themes: 1. Technology 1.1 Easy to use 1.2 Set-up assistance helps 2. Patient Convenience 2.1 Time efficient 2.2 Flexibility 2.3 increased access 3. Patient empowerment to self-manage 3.1 Home environment 3.2 Focus on effective treatment elements 4. Positive therapeutic relationships 4.1 Personal and undivided attention 4.2 Supportive and friendly 5. Adjusting routine treatment 5.1 Modifying usual habits 5.2 Discomfort without hands-on 5.3 Research environment as a safety net) 6. Satisfaction with care 6.1 Satisfaction and enjoyment 6.2 Recommendation to others 7. Patient benefits 7.1 Physical 7.2 Confidence and self-efficacy	Limitations identified by author: Sample constrained to trial participants. Motivation bias. Findings only generalizable to moderate levels of pain and dysfunction characteristic of the sample. Recall bias. Source of funding: Not stated. Evidence gaps and/or recommendations for future research: Future research should determine whether Skype-delivered physical therapy services should be supplemented by an initial in-clinic visit.
Pearson et al.^42^ **Quality score: -**	Research question: Investigated how patients experienced the PhysioDirect service, with the main aim of exploring its acceptability from their point of view. The main objective of this paper is to describe the key variables that determined patient acceptability of the PhysioDirect service and to under- stand how the patient experience differed from those accessing usual physiotherapy care. Theoretical approach: Framework approach Data collection: Telephone semi-structured interviews	Country of study: United Kingdom Population selection: Recruited from RCT Sample selection: Purposive Recruitment approach: Invited to participate Total participants recruited: 82 agreed, 57 interviewed. Mean age of 58. Inclusion criteria: None stated Exclusion criteria: None stated	Method and process of analysis: Framework approach, thematic analysis Key themes: 1. Expectation of the PhysioDirect service 1.1 Physiotherapy is a physical intervention 1.2 PhysioDirect can deliver physiotherapy 1.3 No expectations 2. Physio-Direct as an ‘access point’ into physiotherapy 2.1 Direct access 2.2 Call-back service 2.3 Difficulty in access 2.4 No access 3. Acceptable features of the PhysioDirect service 3.1 Quick and convenient service 3.2 The helpful physiotherapist 3.3 PhysioDirect was effective at providing self management advice 4. Less acceptable features of PhysioDirect 4.1 PhysioDirect was an ‘impersonal’ service 4.2 Communication difficulties	Limitations identified by author: Proportion of participants who agreed to be interviewed from the total invited. 3 participants were interviewed who failed to attend their physiotherapy appointment. Source of funding: None stated Evidence gaps and/or recommendations for future research: None stated
Ryan et al.^43^ **Quality score +**	Research question: (1) to explore the participants in the intervention groups experience of using Action Observation Therapy and to further ascertain the acceptability of this intervention (2) to establish the participants in the control and intervention groups experience of participating in a telehealth study, the motivators for participation and opinions of the trial processes. Theoretical approach: None stated Data collection: Semi-structured interviews conducted online	Country of study: Ireland Population selection: Participants from pilot feasibility RCT on effect of remote Action Observation Theory with Eccentric loading in the treatment of mid-portion Achilles Tendinopathy. Sample selection: Purposive selection Recruitment approach: Follow-up emails sent to pilot RCT participants; all who responded were enrolled. Total participants recruited: 16 participants. Mean age 45 (range 28–58). 10 female, 6 male. Inclusion criteria: Completion of 12 week pilot feasibility study. Exclusion criteria: None stated	Method of analysis: Reflective thematic analysis Key themes: 1. The impact of Achilles Tendinopathy is commonly not prioritised 1.1. The acceptance and minimisation of pain 2. Therapeutic alliance has the greatest impact on support 3. Factors which influenced adherence 4. Action Observation Therapy is valued and recommended 5. Recommendations for future interventions	Limitations identified by author: Positionality and subjectivity of researcher in reflective thematic analysis; participants familiar with online meetings and appointments; interviews relied on retrospective memory; trial researcher led qualitative interviews. Sources of funding: None stated Evidence gaps and/or recommendations for future research: Legitimizing all levels of pain and to encourage individuals with Achilles tendinopathy to action treatment; Action Observation Therapy treatment for persons with Achilles tendinopathy.
Skolasky et al.^48^ **Quality score + **	Research question: Mixed methods research sought to characterize the psychosocial risk, working alliance, and patient-reported outcome (PRO) measures of patients and to describe the experience of patients when taking part in an evidence-based PT program for persons with chronic low back pain (cLBP) delivered by telehealth including concerns that they may have had and advantages and disadvantages that may have encountered. Theoretical approach: None stated Data collection: Telephone-based semi-structured audio-recorded interview.	Country of study: United States Population selection: Selected from parent study who received up to 8 weekly sessions of evidence-based physical therapy using video-enabled computer and broadband internet access, seeking care from at 1 of 3 health care systems (University of Utah or Intermountain Healthcare in Salt Lake City, Utah, or Johns Hopkins Medicine in Baltimore, Maryland. Sample selection: Variation Recruitment approach: Not stated Total participants recruited: 31 participants. Mean age, 42.4 years. 71% female, 29% male. Inclusion criteria: ICD-10 diagnostic code, 18–64 years; at least moderate pain, pain-related disability, cLBP for at least 3 months. Exclusion criteria: Red flags suggesting serious spinal pathology, unable to participate in telehealth, underwent physical therapy for LBP in past 90 days, underwent lumbar spinal surgery in past year, undergoing treatment for substance use disorder.	Method of analysis: Thematic Key themes: 1. Overall experience with telehealth PT 2. Concerns about telehealth PT 3. Advantages of telehealth PT 4. Disadvantages of telehealth PT 5. Recommendations for telehealth PT	Limitations identified by author: Sample not representative of national experience of telehealth physical therapy; participants had access to telehealth technology. Sources of funding: Patient-Centered Outcomes Research Institute (PCORI) (OTS-LBP-2017C1−6486). Evidence gaps and/or recommendations for future research: Health care providers, systems, and policymakers to refine strategies to provide telehealth physical therapy with chronic low back pain, including hybrid approach, and providing necessary equipment, support for patients to use telehealth.
Thesen et al.^47^ **Quality score: ++**	Research question: Mixed methods investigation on the feasibility and potential effects of internet-assisted cognitive behavioral therapy (I-CBT) intervention. The qualitative research examined reasons for participation, experiences with study questionnaires, experiences with the technical platform and intervention content, and eventual gains from the intervention were explored. Theoretical approach: Not stated Data collection: Individual, in-person semi-structured in-dept interviews	Country of study: Norway Population selection: Patients enrolled in the RCT after examination for chest pain at the Cardiac Department at Sørlandet Hospital, Kristiansand, Norway. Sample selection: None stated. Recruitment approach: Examining cardiologists identified eligible patients. Total participants recruited: 10 participants. Mean age of 54. 70% women, 30% men Inclusion criteria: Patients with no cardiac or other obvious somatic diseases that could explain their chest pain symptoms; able to read and speak Norwegian; access to a device with internet connection; physically able to do physical activity. Exclusion criteria: Not stated.	Method and process of analysis: Systematic text condensation (STC) Key themes: 1. External motivation helped the participants overcome the barrier to test the intervention and justify using time on themselves 2. Being involved in the intervention gave insights, tools to cope with pain and the assignments gave them opportunities to test the insights gained 3. Being taken seriously by the health care system, gaining new insights about their own thinking and experiencing the usefulness of physical activity increased internal motivation 4. Gaining insights and prioritizing themselves had wider impacts	Limitations identified by author: The sample size was small, there was no control group, and the patients were recruited in a non-consecutive order. 40% drop-out rate at follow-up, 2 participants refused qualitative interviews. Risk of recall bias. Source of funding: NOK grant from the Norwegian Association for Cognitive Behavioural Therapy Evidence gaps and/or recommendations for future research: Larger scale trials for I-CBT interventions.

a Adapted following NICE guidance on evidence tables for qualitative studies.

Quality scores: ++ All or most of the checklist criteria fulfilled, where they have not been fulfilled the conclusions are very unlikely to alter;  +  Some of the checklist criteria fulfilled, where they have not been fulfilled, or not adequately described, the conclusions are unlikely to alter; – Few or no checklist criteria have been fulfilled and the conclusions are likely or very likely to alter.^49^

### Analysis

The analysis of the included studies was guided by the Consolidated Framework for Implementation Research (CFIR) using deductive thematic synthesis^30^. The CFIR is a tool developed to direct researchers in systematically assessing, evaluating, explaining, and developing the process of integrating evidence-based interventions into health systems and care practices. As a “meta-theoretical” tool, the CFIR distills prevailing concepts and theories from the broader field of implementation science into 5 comprehensive domains and 37 constructs nestled within each domain^50^. These constructs reflect the manifolded factors influencing effective implementation practices and strategies. Importantly, these constructs do not delimit the conditions or interactions required for effective implementation; rather, this tool enables researchers to “select constructs from the CFIR that are most relevant for their particular study setting and use these to guide diagnostic assessments of implementation context, evaluate implementation progress, and help explain findings in research studies or quality improvement initiatives.”^50^

To enhance the transparency of qualitative synthesis,^51^ we focused our analysis on the results sections and relevant tables of the included studies. Themes and patient quotes providing insight into the lived experiences of telehealth were categorized under the CFIR domains and constructs. As we proceeded with the thematic analysis, the data from the included studies condensed around 4 domains and 8 constructs of CFIR (Table 3). We then proceeded to thematically analyze the data captured from the included studies in relation to the meanings of CFIR domains and constructs. This facilitated analysis incorporating the evidence on the patient experience of telehealth in, and the current state knowledge about factors affecting the implementation of health services.^16–18^ In this sense, our analysis considers how the lived experience of telehealth is shaped by individual, systemic, and cultural factors.

**Table 3.** The CFIR and data extraction.

Author, publication year	**CFIR**						
	**I. Interventional characteristics** *The features of an intervention that might influence implementation, specifically the relative advantage of telehealth in terms of its adaptability and its costs as experienced by patients with MSK disorders*			**II. Outer setting** *The features of the external context or environment that might influence the implementation of synchronous telehealth and how the features of these setting influence the implementation of telehealth in the management of MSK disorders*	**III. Inner setting** *The features of the community of practitioners that might influence implementation including how the patient experience of telehealth is shaped by the cultural values, meanings, normal, and practices associated with care for MSK disorders*	**IV. Characteristics of individuals** *The characteristics of patients that might influence how well telehealth is implemented and integrated into their lives. This includes patient attitudes, beliefs, and experiences of telehealth*	
	**I.I Relative advantage** *Patient perception of the advantage of implementing non-pharmacological interventions delivered through synchronous telehealth as compared to in-person care*	**I.II Adaptability** *How non- pharmacological interventions delivered through synchronous telehealth can be adapted, tailored, refined, or reinvented to meet patient needs*	**I.III Cost** *Costs of non-pharmacological interventions delivered through synchronous telehealth and costs associated with implementing telehealth*	**II.I Patient needs and resources** *The extent to which patient needs specific to the use of synchronous telehealth in the non-pharmacological management of musculoskeletal disorders, as well as barriers and facilitators to meet those needs, are accurately known and prioritized by the broader community of practitioners*	**III.I Culture** *Norms, values, and basic assumptions shaping how care for MSK disorders is provided and enacted.*	**IV.I Knowledge and beliefs about the intervention** *Patient's attitudes toward and value placed on receiving non-pharmacological interventions delivered through synchronous telehealth as well as familiarity with facts, truths, and principles related to telehealth*	**IV.II Self-efficacy** *Patient's belief in their own capabilities to participate in the experience of receiving non-pharmacological interventions delivered through synchronous telehealth*
Barton et al.^44^	Theme 1: ‘Telehealth had value, but generally perceived as inferior to in-person care' 1.1 Valuable if in-person care is not an option 1.2 Surprise at value, after initial hesitation Theme 3: ‘Advantages to access safe, expert, and convenient care' 3.1 Facilitates access to the right expertise 3.2 Convenience of not having to travel 3.3 No risk of contracting COVID 3.4 Exercises in home environment *"I realised I don’t always need to see them face-to-face to get value from the consultation and it saves my travel and it saves their time as well. So I think there's a place for it across a range of options for people.” (P2)*	Theme 1: ‘Telehealth had value, but generally perceived as inferior to in-person care' 1.3 Part of hybrid model in certain situations or conditions 1.5 Existing physiotherapist-patient relationships was considered valuable to facilitate telehealth *"I would hope it only became [sic] as an occasional arrangement rather than* *replacing the real thing …. So, it's not something I’d want to do all the time.” (P10)*	Theme 1: ‘Telehealth had value, but generally perceived as inferior to in-person care' 1.4 Cost should be the same or less Theme 2: ‘Challenges related to assessment, diagnosis, ‘hands on’ treatment, observation, communication, and technology' 2.6 Technology concerns *"I was happy to pay for it, but I thought paying the full price when things like rents and the option of [certain] treatment wasn’t available, so I thought a slightly reduced price was pretty reasonable.” (P13)*	Theme 2: ‘Challenges related to assessment, diagnosis, ‘hands on’ treatment, observation, communication, and technology' 2.6 Technology concerns Theme 3: ‘Advantages to access safe, expert, and convenient care’ 3.2 convenience of not having to travel Theme 4. Emotional connection 4. ‘Importance of supportive technology, including video and supplementary resources' 4.1 Video calls important 4.2 Supplemental resources *"This Zoom business is quite complicated.” (P10)*	Theme 2: ‘Challenges related to assessment, diagnosis, ‘hands on’ treatment, observation, communication, and technology' 2.1 Accurate assessment and diagnosis 2.2 Lack of hands-on treatment 2.3 Difficulty with observation 2.5 Perceived as less personal typically but not always *"So if I’ve just go for a run and felt my calf go, I’d need to go to him to assess it for me. So more for a diagnostic level, I’d rather him be present and be able to feel the swelling, feel the injury, feel exactly where it is instead of me trying to point and explain it on video.But now, all I feel like is I’m talking to a screen. You don’t get to feel the energy in the room, you don’t get to feel that person's energy.” (P14)*	Theme 1: ‘Telehealth had value, but generally perceived as inferior to in-person care’ 1.4 Existing physiotherapist-patient relationship was considered valuable to facilitate telehealth *“We’d met [the physio] before on numerous occasions, so I think that made a big difference. I don’t think I’d want to do it to – as the first day effort (P10)”*	
Bell et al.^32^	Theme 1: Convenience *”I was able to step away from my work briefly for my telemedicine visit without having to drive anywhere or wait in the doctor's office." (Patient 5)*	Theme 2: Perceived efficacy Theme 6: Future directions		Theme 5: Feasibility	Theme 2: Perceived efficacy Theme 3: Alterations in the ritual of medicine *“…there are always things that need to be done at home, and I feel less present than when I am in person.” (Patient 3)*	Theme 4: Emotional connection *“During my telemedicine visit, I felt very connected to him [physician], but it was because I already knew him. I feel just as connected to him as in person” (Patient 11)*	
Booth et al.^33^	Theme 1: The vPMP is entirely acceptable 1.1 Learning to self-manage persistent pain whilst at home 1.2 Receiving a high quality of care from home. 1.3 Enhancing the potential of rehabilitation using technology 1.4 Enabling pain management *“I would recommend it yes, a hundred per cent, no question… I made changes around the home and I can see it every day and it reminds me every day to actively think about what we were learning on the course.”*	Theme 1: The vPMP is entirely acceptable 1.5 Overcoming social distancing requirements of COVID-19 through technology Theme 2: The vPMP is not acceptable at all 2.1 Inappropriate home environment for virtual therapy *“I mean because of lockdown if that course hadn’t been virtually… I don’t think we would have had our chance up until the end of next year or something knowing what the situation is at the moment in hospital and that there is a back log so it was good in that sense.”*		Theme 2: The vPMP is not acceptable at all 2.3 Technological issues *“My internet was such a pain throughout. I felt like I missed parts of the sessions because I had to keep logging back in and I found that practical element really challenging and obviously no one can really do anything about it.”*	Theme 2: The vPMP is not acceptable at all 2.2 Communication challenges with virtually delivered care 2.4 Concerns about the quality of remotely delivered care *“For me I found it difficult with the physio not being face-to-face. The sessions were really helpful, but I just felt like I needed it more in person, especially because since I have seen my physio in person and I have been referred for a few other things because my joints were really bad but from home you couldn’t really tell that and when we should be doing something differently.”*		Theme 1: The vPMP is entirely acceptable 1.1 Learning to self-manage persistent pain whilst at home. 1.6 Virtual peer support *“[Learning to self-manage] has improved my quality of life and given me a bit of hope which is what I didn’t have before I started the course. I didn’t have these methods or a way of dealing with stuff which I now have, and some ideas of what I can try and things that can help that's valuable.”*
Cottrell et al.^45^	Theme 1. Experience of telehealth was overall positive 1.1 Grateful for the opportunity to continue access to care during COVID-19 restrictions 1.2 Telehealth service provided convenience, reduced waiting and travel time and reduced cost 1.3 Avoiding hospital when immunocom promised reduced anxiety and supported feeling of safety during COVID restrictions *"Eliminated the need to go and visit the hospital – [I’m] a vulnerable patient with cancer history so have to be particularly careful with exposure.” (P18)*	Theme 2: A blended model of telehealth and in-person consults is preferred 2.1 Preferred mode of delivery is dependent on appointment type or nature of tasks required for that appointment 2.2 Having the choice of a mix of in-person and telehealth appointments is preferred *"I would like to have some of my services via telehealth and some face to face when required.” (P3)*	1. Experience of telehealth was overall positive 1.2 Telehealth service provided convenience, reduced waiting and travel time and reduced cost *“On top of the fact that I don’t have to travel 250 km round trip and don’t have to pay $80 in parking fees for a short appointment. Improved my mental health in many ways’.” (P1)*	Theme 3: Technology 3.1 Telehealth platform was easy to use 3.2 Inconsistent telehealth connection impacted experience Theme 4: Support is required to engage in telehealth 4.1 More technology support is required 4.2 Patient support is required to engage with telehealth 4.3 Patient support is required to prepare for and engage in telehealth consults *"Needs better tech[nical] support on the hospital end.” (P23)*			
Dharmasri et al.^46^	Theme 4: Intervention delivery			Theme 4: Intervention delivery *“I don’t have a computer and I don’t know how to work them.”*			
Ernstzen et al.^34^	Theme 3: The online learning environment *“It was different in terms of where you individually have people that have to go to a hospital, people who have to travel, didn’t have to go out of their space to be part of it. So that to me was amazing so I didn’t find that at all challenging.” Gr3_P6*	Theme 3: The online learning environment *“It was very nice but sometimes it was a little bit of a challenge get- ting the kids not to make a noise, so some days – my mom stays around the corner from me – so when I know it is busy by me, I will go to my mom's house just to get quietness sometimes, but it was very nice – I enjoyed it.” Gr2_P5*	Theme 3: The online learning environment	Theme 3: The online learning environment *“I think when they do this with people and especially with older people, they need to …I think they should explain to them just send them a little video, just explain to them?”. Gr3_P6*			Theme 1: The journey of discovery Theme 2: Building peer and therapeutic relationships *“Every week he would – before we started the program – he gave us a platform to basically speak about how we feel, how we are dealing with our pain, how we are dealing with our thoughts, so he gave us the, ja – he gave us a platform for us to be heard.” Gr1_P4*
Ezzat et al.,^35^	Theme 1: Participant perceptions of telehealth acceptability was highly influenced by exposure 1.1 Telehealth considered easy, convenient, and flexible by telehealth group, but inferior and misunderstood by in-person group *“I think the fact that it allows people to do it in their own home and I had the flexibility with that because of my job and being a shift-worker.” P9*	Theme 1: Participant perceptions of telehealth acceptability was highly influenced by exposure 1.2 Telehealth was well supervised and quality feedback was provided *“The physios were really onto anything that they gave me a lot of feedback as to my positioning, and it was very detailed, and it seemed to me that it was like they were there in the room. So, that worked very well.” P19*		Theme 1: Participant perceptions of telehealth acceptability was highly influenced by exposure 1.3 Technology hiccups could be worked out *“It was a bit annoying with the technology didn't work but I mean that's part of life but, otherwise, I actually quite enjoyed [telehealth].” P3*			2. Telehealth and in-person reported similar benefits of program 2.1 Reductions in fear of pain and joint damage resulting in improved confidence to engage in physical activity and exercise 2.2 Changed beliefs on importance and value of exercise 2.3 Self-reported improvements in pain and function *“Made me realise I have to be more active and more specifically active, not get up and try and run around the block five times, but do things that are going to help strengthen the knee.” P2*
Fraser et al.^36^	Theme 2: Expectations and reflections 2.3 Reflections on receiving the intervention				Theme 2: Expectations and reflections 2.3 Reflections on receiving the intervention *“I don’t think it would really be a long-term answer, but it's certainly helpful in the interim, yes … it's really because you can’t actually see someone and you’re not sitting in the same room as someone.” (IV5)*	Theme 2: Expectations and reflections 2.3 Reflections on receiving the intervention	
Gilbert et al.^37^	Theme 1: Situation of care 1.3 The availability of healthcare to the patient Theme 2: Expectations of care 2.2 Psychological status Theme 3: Demands on the patient 3.2 Social demands "*One of the reasons why the screens would be good is I would feel less anxious to talk to someone through a screen, but I would in the same room."(P9)*			Theme 3: Demands on the patient 3.3 Consequence of choice Theme 4: Capacity to allocate resources to care 4.2 Infrastructure *"For me, it's the equipment. I only live in a small—and it is small, isn't it—a small two-bedroom house. I would have nowhere to store the equipment… there's no option out there to rent equipment.” (P19)*	Theme 2: Expectations of care 2.1 Desire for contact *“If it's something simple then, yes, that's a good idea. If it's something a bit more complicated they actually have to come and see it because it's more of a hands-on type of thing.” (P8)*	Theme 1: Situation of care 1.1 Clinical status Theme 2: Expectations of care *“If I'm having a flare-up, sometimes I can't even leave the house. I get stuck indoors and I just wouldn't be able to do much really.” (P7)*	Theme 2: Expectations of care 2.2 Psychological status Theme 3: Demands on the patient 3.2 Social demands *“You don't like the way that your life's going to look because you know you're not going to be able to achieve all the things that you want to achieve.” (P17)*
Gilbert et al.^38^	Theme 1: The context for the consultation 1.3 Congruence Theme 2: The implementation process of VC 2.2 Cognitive participation *“For me, the difference between virtual and face-to- face is a big three hour difference of time.* *I can have my virtual appointment over* *the phone wherever I am, set up and go with it, and be done within half an hour, 45 minutes.” [P4-6]*	Theme 2: The implementation process of VC 2.3 Collective action Theme 3: Results 3.2 How Preferences for VC are decided *“You're reliant on someone's ability to be able to use the technology before you even* *get to know them.” [P4-6]*			Theme 1: The context for the consultation 1.1 Normative expectations 1.2 Relational expectations 1.3 Congruence 1.4 Potential Theme 2: The implementation process of VC 2.1 Coherence *“No, I think it's far more effective to have face-to-face. They do as best they can, but there's limitations to having a 2D camera and being able to see in 3D, which obviously we see in 3D.” [P4-2]*	Theme 1: The context for the consultation 1.2 Relational expectations *“As I say, if I had only ever had virtual physio, so I'd seen the physio once in* *hospital and all the others were virtual, I wouldn't have felt as close.” [P-14]*	
Hasani et al.^39^	Theme 1: Acceptability of the telehealth sessions and gym-based exercise intervention Theme 2: Enablers of adherence with telehealth monitoring 2.1 Usability of the software 2.2 Flexibility in arranging an appointment time *“I did not have to go and wait in an office or what- ever. I could just go to the gym and just get started and if he was not ready or whatever then I could just get into my workout and he [physiotherapist] would join me halfway through sometimes. There was no waiting around.” (P4)*	Theme 1: Acceptability of the telehealth sessions and gym-based exercise intervention *“If you do not need actual manipulation, like a massage or something like that, or physical touching of the area, then that would be fine.” (P 7)*		Theme 3: Barriers to adherence with telehealth monitoring *“It was the longer regimen that I was on [low-intensity group]. A couple of times my battery went out and I was using up all my data because it was an hour and a half session.” (P 8)*	Theme 2: Enablers of adherence with telehealth monitoring 2.3 Therapeutic alliance *“I guess, there is a certain amount of hands-on with the physio, but depending on the type of issues that someone has, there is certainly scope for being able to treat them from afar, once you have got an accurate idea of what you want to do with them.” (P 8)*		Theme 2: Enablers of adherence with 2.3 Therapeutic alliance Theme 4: Enablers to adherence with the exercise interventions 4.1 Regular contact Theme 5: Barriers to adherence with the exercise interventions 5.1 Time *“I think if you did not have to talk to anybody, you might get lazy some days and not bother. Whereas that sort of kept you committed and making sure you will not forget your exercises.” (P 6)*
Hinman et al.^40^	Theme 3: Program structure				Theme 3: Program structure *“With the knee, I suppose I was imagining there’d be more a hands-on assessment of my knee, and it was just really ‘How are you going with the exercises?’ ‘Are you doing them?’ ‘Aren’t you, and how can you do it so it's not as painful?’” (Mary)*		Theme 1: Genuine Interest and Collaboration Theme 2: Information and accountability *“The fact that they were genuinely, or seemed genuinely, interested and were monitoring your progress, you know what I mean, and you sort of go back to your health coach, for example, and you feel a little bit proud that you’ve achieved what you, what's been set for you, to achieve, and similarly with the physio [physical therapist], you know, I’d say ‘I’ve done it’ with a big smile on my face.” (Tom)*
Hinman et al.^41^	Theme 2: Convenience *“I could be in different places to use it, I wasn’t having to take the time out of the day to go to somewhere or to find a set position, so I was able to Skype from work.” (Lois)*			Theme 1: Technology *“We had to sort of repeat things a couple of times. But in general, it worked, worked extremely well. I think the only negative was there were times when the transmission was not great. So, I thought it was very, it was very positive.” (Nathan)*			Theme 3: Patient empowerment to self-manage Theme 4: Positive therapeutic relationships 4.1 Personal undivided attention *“I think with the Skype physio, it's more individualized and very much you. You know it was more concentrated.” (Evelyn)*
Pearson et al.^42^	Theme 3: Acceptable features of the PhysioDirect service 3.1 Quick and convenient service *“It was quick. That was the, um, it seemed to plug the gap of having to wait for an appointment.” (Peter)*	Theme 2: PhysioDirect as an ‘access point’ into physiotherapy 2.1 Direct access *“I got through alright, there was no problem getting through.” (Walter)*			Theme 1: Expectations of the PhysioDirect Service Theme 4: Less acceptable features of PhysioDirect 4.2 Communication difficulties *“Yeah, I found it a bit, quite difficult, because it's hard to explain isn’t it, even, not just on the phone but to anybody. I mean, the pain I was in was really, really bad, so, um, I would have preferred to have saw somebody…” (Jenny)*	Theme 4: Less acceptable features of PhysioDirect 4.1 PhysioDirect was an ‘impersonal’ service *“Because it's a face-to-face, personal thing. You know that there's somebody sitting there waiting for you turn up and you don’t or you’re canceling your appointment that somebody's gone to the trouble to make for you, whereas a phone call's just a phone call and it can be anytime and anywhere, so, it's less personal.” (Hannah)*	Theme 3: Acceptable features of the PhysioDirect service 3.3 PhysioDirect was effective at providing self- management advice *“It's a good thing because obviously, not everybody knows the best way in order to aid their injury. When I hurt my ankle and they sent out the information to me after the initial over the phone consultation with the PhysioDirect, they sent me out a book of all the different exercises in order to aid my ankle.” (Robert)*
Ryan et al.^43^	Theme 2: Therapeutic alliance has the greatest impact on support *"I felt honest to God I could pick up the phone or* *email you at the drop of a hat if I ever needed to."*					Theme 2: Therapeutic alliance has the greatest impact on support *“I did not feel at any time kind of isolated, I felt the support was always there.” (P2).*	Theme 3: Factors which influenced adherence *“I think if we hadn’t had those check-ins, if we just had the one at the start and the one at the end prob- ably my compliance and commitment wouldn’t have been as much as it was.” (P2)*
Skolasky et al.^48^	Theme 1: Overall experience with telehealth PT Theme 3: Advantages of telehealth PT *“[I] didn’t have to arrange for childcare to have an appointment” (P 3628–64)*		Theme 1: Overall experience with telehealth PT *“Marked time savings, more convenient faster service, cost savings” (P 3628–379).*		Theme 1: Overall experience with telehealth PT Theme 2: Concerns about telehealth PT Theme 4: Disadvantages of telehealth PT *“The only thing about in-person physical therapy is they can put their hands on you and actually physically show you how to do the exercise, and with the telehealth they can't put their hands on you, but they can explain it better” (P 3629–45).*	Theme 2: Concerns about telehealth PT Theme 4: Disadvantages of telehealth PT *“It's harder to make personal connections when it's just on a screen” (P 3628–118).*	Theme 1: Overall experience with telehealth PT * “I asked about in person because once he started explaining to me how this was going to work, I could tell that it wasn’t going to be for me. So maybe if it was done in a better way* *—I need to be in person to be able to do these things with my back situation” (P 3629–77).*
Thesen et al.^47^							Theme 1: Overcoming barriers for participation Theme 3: From external to internal motivation *"That phone call, I almost looked forward to it, because it, ehm, committed me more. And had [the call] not happened during those weeks, then…it could have been, I am just saying (draws breath), a bit so and so.” (Aleksander)*

Given the intentional and intrinsic flexibility of CFIR,^52^ we modified the grammar of the relevant domains to better suit the scale and focus of the studies we analyzed (please see Appendix B).

We used GRADE-CERQual (Confidence in the Evidence from Reviews of Qualitative research) to assess confidence in the synthesis findings^53^. The assessment is based on four key components: (1) methodological limitations of included studies; (2) coherence of the review finding; (3) adequacy of the data contributing to a review finding; and (4) relevance of the included studies to the review question. The included articles were reviewed by the authors, reaching consensus as required.

## Results

### Study selection

**Figure 1.**
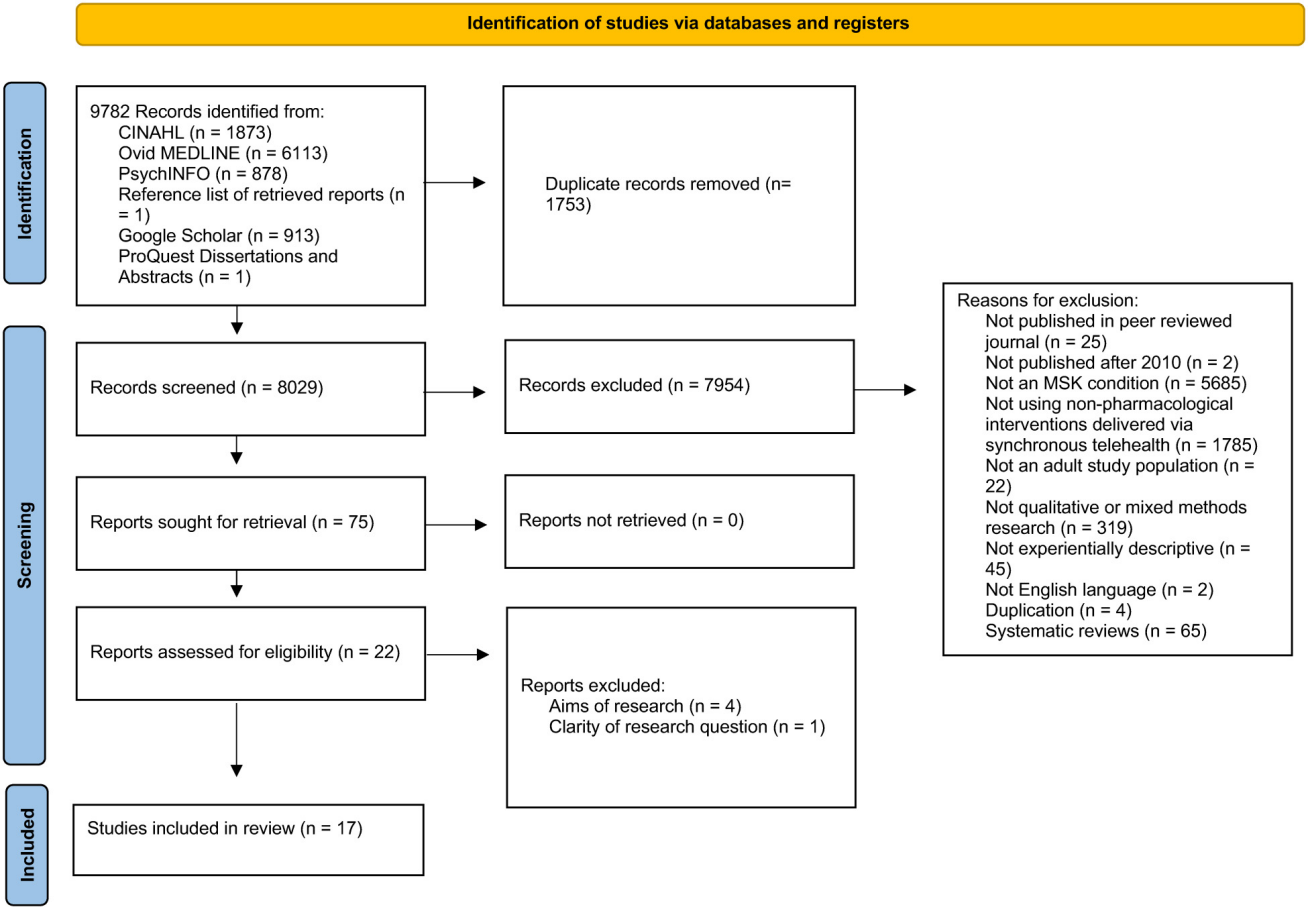
PRISMA diagram summarizing flow of included studies.

Source: Page MJ, McKenzie JE, Bossuyt PM, Boutron I, Hoffmann TC, Mulrow CD, et al. The PRISMA 2020 statement: an updated guideline for reporting systematic reviews. BMJ 2021;372:n71. doi: 10.1136/bmj.n71. For more information, visit: http://www.prisma-statement.org/.

We identified 9782 references, screened 8029 references, and critically appraised 22 articles (Figure 1). A total of 5 articles were excluded on critical appraisal, resulting in the inclusion of 17 studies in our review. Twelve of the included studies were assessed using the CASP tool^32–37,39–43^ and five using the MMAT tool^44–48^. During the screening process a 98% absolute agreement was met, with a Kappa score of 0.99 based on a 10% sample.

### Study characteristics

Eight of the included studies were conducted before the declaration of the global COVID-19 pandemic in March 2020,^35,36,39–42,46,47^ and nine studies afterwards.^32–34,37,38,44,45,48^ One study was conducted in Norway,^47^ one in Ireland,^43^ one in South Africa,^34^ three in the United States,^32,46,48^ five in the United Kingdom,^33,36–38,42^ and six studies were conducted in Australia.^35,39–41,44,45^ Nine of the included studies recruited participants from a previous or ongoing clinical trials.^35,36,39–43,46,47^

Practitioners represented in these studies included mental health professionals, occupational therapists, physicians, and physiotherapists. Non-pharmacological interventions for MSK disorders were delivered via telephone conferencing^36,40,42,44,46,47^ and videoconferencing using applications such as Zoom, Skype, and Microsoft teams.^32,33,37,39,41,45^ Six studies reflected the use of telehealth within publicly funded hospitals,^33,36–38,42,45,47^ four from academic hospitals,^34,35,43,48^ two from private physiotherapy practices,^40,44^ one from both private and public physiotherapy practices,^39^ one from a private medical practice,^32^ and one study reflected the use of telehealth in both a not-for-profit hospital and within a veterans healthcare system.^46^

### CFIR domains and constructs

We identified four domains and their respective constructs from the CFIR that were most relevant for both synthesizing and analyzing the data about the patient experience with telehealth. We summarize these results, organized by each of the four CFIR domains and their respective constructs (see Table 3 for full definitions of each domain and construct).

### Domain I: Interventional characteristics

This domain refers to the features of an intervention that might influence implementation including its adaptability and its costs as experienced by patients with MSK disorders.

#### Construct I.I: Relative advantage

Across the included studies there was consistent reporting that patients appreciated the convenience, efficiency, flexibility, and reduced travel times associated with telehealth.^32–35,37,39,41,42,45,46,48^ Not having to schedule childcare or take time away from work was seen as an advantage by many of the patients involved in the included studies. Studies conducted during the pandemic highlighted the advantage of telehealth in preventing the transmission of COVID-19, and in ensuring continuity of care during lockdowns.^32,44,45^

In terms of the experience of pain, patients who participated in the Hinman et al.^41^ study made the poignant observation that telehealth is particularly advantageous for those in acute pain. With telehealth, patients did not have to travel in pain, navigate parking lots and clinical buildings, or dwell uncomfortably in waiting rooms. Similarly, several studies examining virtual chronic pain management programs found that telehealth offers high-quality care to people living with chronic pain whose symptoms such as fatigue and anxiety have prevented them from participating in in-person appointments.^33,48^

Indeed, prior to the pandemic, Gilbert et al.^37^ found that some patients felt that videoconferencing relieved the anxiety associated with face-to-face interactions in an orthopedic rehabilitation setting. These sentiments were echoed in the work of Fraser et al.^36^ and Booth et al.,^33^ with both studies finding that patients liked telehealth-based interventions because it enhanced feelings of anonymity, reduced stigma, and created conditions for communication without judgment.

#### Construct I.II: Adaptability

The experience of adapting to the remote delivery of MSK care was varied throughout the included studies. Several studies conducted before the pandemic reported that patients considered exercise instruction, coaching, and pain focused counseling interventions adaptable to telehealth.^32,39,44,45^ As Ezzat et al.^40^ describe, patients felt telehealth was acceptable and adaptable to their MSK health needs once they had been exposed to and experienced this form of care. Nevertheless, patients in all of these studies posited that telehealth was not adaptable for urgent complaints, complex cases, or MSK issues they believed necessitate an in-person exam.^32,44,45^

Limitations in adapting MSK care to telehealth were also articulated in studies conducted during the pandemic. Bell et al.^32^ found that patients did not want to receive serious news via telehealth. Other studies reported that patients had difficulty adapting to the telehealth format because of distractions in their home-environment^34^. Barton et al.^44^ identified that patients were better able to adapt to telehealth at home provided they had an existing relationship with their physiotherapist.

Critically, Gilbert et al.^37,38^ and Booth et al.^33^ point to the specific set of challenges experienced by people with disabilities when adapting to telehealth, which include sensory issues, communication and literacy barriers, and access to and use of specialized equipment and technology to facilitate in-home care.

These findings suggest that it is not only a matter of exposure to telehealth that aids in its adoption in the non-pharmacological management of MSK disorders; rather, it is the conditions in which one is exposed to telehealth that matter for how this format is perceived as adaptable.

#### Construct I.III: Cost

One salient issue identified was a perceived pricing disparity between telehealth and in-person care. Barton et al.^44^ found that some patients thought telehealth should cost the same or less than in-person care. These patients expressed concerns about the accuracy of telehealth as a format for assessing and diagnosing MSK disorders and did not feel that telehealth encounters had the same value as in-person appointments.^44^ Other studies noted the financial and energetic advantages of telehealth, including not having to spend money on travel expenses and not losing income to accommodate for appointments, were valued by patients.^34,37,39,45,48^

### Domain II: Outer setting

This domain concerns the external contexts and conditions in which patients experience telehealth and the effects of those situations on the telehealth experience.

#### Construct II.I Patient Needs and Resources

Across the included studies, patients consistently described a set of interconnected needs that point to broader structural issues and resource constraints affecting the experience of telehealth. Those needs were primarily related to the access to smartphones, devices, or computer for telehealth appointments, and the availability of reliable, affordable, and secure internet.^32–35,37–39,41,44–46^

Several studies raised concerns about digital literacy^34,35^ and the educational needs that must be addressed for all patients to meaningfully engage with telehealth. Dharmasri et al.^46^ found that many older African American adults included in their study on telehealth interventions for osteoarthritis either did not own a computer or did not believe they had the skills to manage a telehealth appointment. Hasani et al.^39^ found that many patients needed guidance on camera set-up during supervised exercise training via telehealth. Cottrell et al.^45^ and Barton et al.^44^ suggest the need for adequate appointment preparation and support for telehealth including help with setting expectations and payment.^25^ Indeed, Ezzat et al.^40^ found that technological barriers to telehealth were overcome with planned time for training and practice, further gesturing the importance of assessing for the technological and infrastructural barriers patients face before engaging in telehealth based care.

### Domain III: Inner setting

In this domain we explore how the cultural values, meanings, and norms associated with MSK care are contested and renegotiated through the experience of telehealth.

#### Construct III.I: Culture

The implementation of telehealth in the treatment of MSK disorders prior to and during the pandemic challenged beliefs that patients hold closely about how care ought to be delivered. Across the included studies, patients described the in-person physical exam as playing a critical role in assessing and treating MSK disorders. Patients expressed concern regarding the validity of telehealth assessments, wondering openly if their practitioners could diagnose without physical touch.^32,33,36–40,44,48^ Several studies noted that patients struggled to explain their experience of pain or their MSK complaints during a telehealth encounter.^32,42,44^

These feelings of distance and disconnect were especially acute in several studies conducted during the pandemic.^32,33,37,44,45,48^ As one patient in the Barton et al.’s^44^ study put it, “But now, all I feel like is I’m talking to a screen. You don’t get to feel the energy in the room, you don’t get to feel that person's energy (P14).” Bell et al.^32^ suggested the removal of the physical exam during the turn to telehealth represented a profound “Alteration in the ritual of medicine” that left many patients feeling as though “Something [was] missing” from their appointments. In discussing their findings, these researchers suggested that the change in the expected and familiar practices of consultation left patients feeling less fulfilled with their telehealth encounters. These results question the lasting effects of the implementation of telehealth during the pandemic on the rituals of medical encounters and how patients may have been affected by this disruption to the ceremonial practices they value so deeply. Many patients also expressed a desire for a hybrid model of care, envisioned as including an in-person initial evaluation, followed by telehealth appointments.^34,48^

Some studies also reported that patients were concerned about how telehealth would impact their ability to develop a relationship with a new practitioner.^40^ In contrast, one study reported that some patients felt “A subtle shift in power” when consulting with their providers remotely. This was felt in situations where patients received undivided attention from their physical therapist because of telehealth, thereby feeling they were the focus of the encounter.^41^

### Domain IV: Characteristics of individuals

This domain elucidates how patient attitudes, beliefs, and experiences influence the implementation of telehealth into MSK care.

#### Construct IV.I: Knowledge and beliefs about the intervention

In the included studies, we find immense variance in patients’ knowledge and beliefs about the use and value of telehealth. Gilbert et al.^37,38^ offered the important reminder that patients arrived into the telehealth encounter with a set of “relational expectations” which reflected “normative conventions” around how patients and providers related to one another in the context of MSK care. In their study on the PhysioDirect service in the NHS, Pearson et al.^42^ reported that some patients perceived the telephone-based intervention as an impersonal format that made it difficult to forge a connection with their practitioner. Pearson et al. describe that this experience contributed to the perception of the service as being less valuable than face-to-face consultations.^42^

Several of the included studies described that patients’ perception of telehealth is influenced by the quality of the therapeutic alliance they have with their MSK health provider. These studies elucidate that if a patient feels supported by this relationship, and if patients are collaboratively engaged in their care, they will think favorably of telehealth.^34,37,38,43^

#### Construct IV.II: Self-efficacy

Research conducted before the pandemic suggests that the home environment plays a role in how connected patients feel in their telehealth encounters.^39^ Some of the included studies reported that patients experienced enhanced feelings of comfort working with their practitioners at home which in turn facilitated increased adherence to home exercise programs, changed perceptions of the value of exercise, and self-reported improvements in pain and function.^35,38,41,43^ On the other hand, some studies described the difficulties patients had in learning exercises remotely, suggesting hands on exercise correction was needed.^48^

Several studies described competing demands on patients while at home, including work and caregiving responsibilities, making it difficult for patients to be present for a telehealth appointment or adhere to their prescribed exercises.^32,37^ Further, Gilbert et al.^37^ identified that virtual consultations can provoke anxiety and diminish the experience of the intervention because the format exposes the private sphere of the home. These barriers to self-efficacy and adherence cast the relative advantages of telehealth in a new light. While telehealth may relieve patients of the labor of travel, and allows for easier scheduling, it also brings the encounter of MSK management in the home—a social space not always designed to hold for such medical consultations.

Amplifying results about the importance of the patient–practitioner relationships, studies demonstrated that patients experience deeper feelings of engagement with their care when they have an existing relationship with their practitioner.^39–41^ With a secure therapeutic alliance in place, telehealth encounters become motivating for patients, enabling commitments to exercise prescription and self-management.^39–41^ When this alliance is not in place, patients lack accountability and support,^40^ and face emotional barriers to committing to their plan of management.^47^

Similarly, the emergence of supportive relationships between patients was reported in studies that involved group-based telehealth interventions for chronic and persistent pain. Studies by Ernstzen et al.,^34^ and Booth et al.,^33^ report that the virtual setting propagated forms of peer support and relationships that patients maintained beyond the intervention sessions, suggesting that telehealth interventions have the potential to positively impact the rehabilitation experiences of individual and communities.

### Confidence in review findings

We assessed the findings arising from collected data against each of CFIR domains and constructs using GRADE-CERQual^54^ and assigned a confidence rating of no or very minor concern, minor concern, moderate concern, or serious concern (Table 4). We identified minor concerns with each domain and construct, except for *Construct I.I Relative Advantage* which posed moderate concern. Moderate concern was expressed because of methodological issues pertaining to three mixed methods studies included did not clearly report on the integration of their qualitative and quantitative data^44,45,48^ and one study did not provide adequate consideration of the patient–researcher relationship.^35^ The remaining identified minor concerns reflect issues with appropriate research design,^32,46^ and with results sections of studies focused more heavily on program delivery than patient experience.^36,40,42,46^ We recognize that some of the concerns we identified may reflect challenges pertaining to the publication of qualitative and mixed methods health research, and constraints faced by teams conducting research during the COVID-19 pandemic. We believe our findings reasonably represent our phenomena of interest,^54^ and have assigned an overall confidence rating of moderate for the findings of our review.

**Table 4.** GRADE CERQual confidence in review findings.

Summary of review finding	Studies contributing to the review finding	Methodological limitations	Coherence	Adequacy	Relevance	CERQual assessment of confidence in the evidence	Explanation of CERQual assessment
**CFIR Domain I: Interventional characteristics**							
**Construct I.I Relative advantage**	^32–42,44,46,48^	Moderate concern	Minor concern	Moderate concern	Minor concern	Moderate concern	Moderate concern reflects methodological limitations pertaining to mixed methods integration in two studies^44,45,48^ and inadequate consideration of patient–researcher relationship^35^, level of experiential data reflected in one study^46^, and richness and relevance of experiential data in^32,40,46^
**Construct I.II Adaptability**	^32–35,39,42,44,45^	Moderate concern	No concern	Minor concern	No concern	Minor concern	Moderate concern reflects methodological limitations pertaining to mixed methods integration in two studies^44,45^ and inadequate consideration of patient–researcher relationship^35^, level of experiential data reflected in one study^46^
**Construct I.III Cost**	^34,44,45,48^	Moderate concern	No concern	No concern	No concern	Minor concern	Minor concern reflects methodological limitations pertaining to mixed methods integration in two studies^44,45,48^
**CFIR Domain II: Outer setting**							
**Construct II.I Patient needs and resources**	^32–35,37,39,41,44–46^	Moderate concern	No concern	Minor concern	Minor concern	Minor concern	Minor concern reflects methodological limitations pertaining to mixed methods integration in two studies^44,45^ and adequate consideration of patient–researcher relationship^35^, richness of experiential data in one study^26^, and adequacy of analysis in two studies^32,40^
**CFIR Domain III: Inner setting**							
**Construct III.I Culture**	^32,33,36,37,39,40,42,44,48^	Moderate concern	No concern	Minor concern	Minor concern	Minor concern	Minor concern reflects methodological limitations pertaining to mixed methods integration of two studies^44,48^, adequacy and relevance of data analysis in two studies^32,40^
**Domain IV: Characteristics of the individual**							
**Construct IV.I Knowledge and beliefs about the intervention**	^32,33,36,37,42–44^	Moderate concern	No concern	No concern	Minor concern	Minor concern	Minor concern reflects methodological limitations pertaining to mixed methods integration of two studies^44,48^, adequacy and relevance of data analysis in two studies ^32,36^
**Construct IV.II Self-efficacy**	^33–35,37,39–43,47^	Minor concern	No concern	No concern	Minor concern	Minor concern	Minor concern reflects methodological limitations pertaining to mixed methods integration of one study^48^, minor concern reflects adequate consideration of patient–researcher relationship^35^, the process of data analysis in one study^41^, lack of positionality statement in one study^42^, adequacy and relevance of data analysis in two studies^36,41^

## Discussion

Our review adapted the CFIR to examine the contextual and experiential factors shaping the implementation of synchronous telehealth in the non-pharmacological management of MSK disorders. Using CFIR, we identified four key domains shaping the lived experience of telehealth from the patients’ perspective: (1) Interventional Characteristics, (2) Outer Setting, (3) Inner Setting, and (4) Characteristics of Individuals. Each of these domains and their associated constructs have bearing on how we understand the ongoing implementation of telehealth. Broadly, our findings demonstrate that the experience of telehealth is influenced by access and comfort with technology, the environments in which telehealth is lived, and existing patient attitudes, beliefs, values, and experiential knowledge about what constitutes good care in the broader field of MSK Health. These findings are also consistent with recent commentaries on the use of telehealth in manual therapy practice.^15^ Using GRADE CERQual we assigned a moderate level of confidence in our findings.

Recent discussions on rapid reviews and evidence synthesis during the COVID-19 pandemic have gestured to the importance of developing practical implications that support how practitioners and decision makers grapple with information on emergent phenomena.^55^ We have developed a set of implications which leverage social theory to understand how our findings may have been contoured by broader cultural, political, historical dimensions that also factor into experiences patients have with telehealth. These implications are meant to help appreciate the complexity of patient experiences while also provoking novel forms of inquiry and action in this emergent area of implementation.

### Implication I: Relationships matter

Our review found that telehealth is experienced positively when adapted into situations where there is an existing patient–practitioner relationship. These findings suggest that the experience of telehealth is deeply influenced by the knowledge and beliefs patients hold about their relationships with their providers. When patients have positive telehealth experiences, it appears to be due to a felt sense of connection with their provider, or because their providers demonstrated an effort to forge a connection through the distance.^39–41^ This is an important consideration for the use of telehealth in the management of acute MSK conditions with no prior onset, or previous history of being treated by an allied health provider. The ongoing use of telehealth in MSK care must consider how these technologies can be used to create and sustain patient–provider relationships.

### Implication II: Transforming the ritual of medicine

One of the studies included in this review contended that telehealth and the virtual physical exam poses an “alteration in the ritual of medicine.”^32^ Bell et al.^32^ suggest that the physical exam is a kind of ceremony—a way of making and demonstrating a shared commitment to care and rehabilitation. Hands-on care is thus critical for patients in developing relationships with their providers. Patients place immense value in being able to feel that their practitioner is attuning to their injury through the act of the physical exam. Our review also demonstrates that the absence of the physical exam negatively influences the patient experience of telehealth.^32,36,37,40^ When we think about the continued implementation of telehealth in MSK, especially in situations where physical exams cannot be performed, consideration should be given to what new rituals could be adapted to the encounter that might help to forge bonds of trust and connection.

### Implication III: Technology continues to be barrier to telehealth

As demonstrated by our results, the continued implementation of telehealth in the broader field of MSK care should support how patients access and utilize telehealth technologies. Other reviews evaluating the implementation of telehealth technologies highlight how inequalities concerning internet and device access continue to influence the experience of telehealth.^10,56^ These reviews suggest that the potential of telehealth to expand the reach of medical services is contingent on investments in telecommunications infrastructure and on the broader recognition of the socioeconomic inequalities that shape how people access telehealth.^57–59^

Social scientists have identified that telehealth technologies have primarily imagined clinicians, not patients, as their primary users.^60,61^ There is an opportunity for patients, particularly persons with disabilities, to be involved as stakeholders in the continued implementation of telehealth, and be equipped with the necessary training and tools to determine how these technologies are used in their care.

### Implication IV: Telehealth is work for patients

In their recent systematic review on the use of virtual consultation in orthopedic rehabilitation, Gilbert et al.,^11^ argue that “The use of virtual consultations changes the work of being a patient.” They contend that virtual consultations catalyze new forms of “self-management” for patients. We uncovered similar results in our review, with included studies reporting that patients using telehealth must learn to be their own educators and technical support staff^37,44,45^ and take on the work of transforming their home environments into spaces conducive to telehealth encounters.^37,44^

Furthermore, the movement of healthcare into the home has implications for the patient experience. Sociologists refer to this phenomenon as “deinstitutionalization”—a term which names the mobilization of technology to allow clinical encounters to occur outside of conventional medical environments.^62^ Patients bear the brunt of the work associated with the deinstitutionalization of MSK care and, as Gilbert et al.,^11^ suggest, this may determine “patient preference for or against virtual consultations.” More research is needed to understand the role of the home and homelife in the experience of telehealth. Insight into housing security, perceptions of safety, and experiences of home-based violence will help us understand when, where, and how telehealth can be used or ought to be used.

### Implication V: A new type of patient

The process of deinstitutionalizing MSK rehabilitation vis-a-vis the implementation of telehealth has far reaching effects beyond transforming peoples’ homes into makeshift clinical spaces. It also has an impact on how people relate to and enact the role of the patient. The studies included in our review evidence that telehealth is transforming the concept of “patienthood”—thus suggesting that being a patient is not a fixed identity category but rather an ongoing process of interactions with different types of medical providers in different medical environments that transform people into patients.^63–66^ How people take on and perform the patient identity reflects their expectations, behaviors, situated experiences, and formations of power that are shaped by interactions with medicine and medical providers. In this sense, we can consider that part of the strain described by patients reflects learning how to enact the patient role in the home environment where people with MSK disorders may not be used to acting as patients. The continued implementation of telehealth needs to consider that patients are actively navigating and negotiating these new challenges to their identity as they receive care at a distance.

### Strengths and limitations

Strengths of our review include the use of consistent, transparent methods to screen, critically appraise, and extract data from included studies, and robust quality assurance processes. We also worked closely with an experienced health librarian to conduct our literature search.

There are limitations shaping our review. We only included studies published in English, and this may have excluded relevant studies. We believe this is an unlikely source of bias.^67^ Our use of rapid review methodology meant that one reviewer was responsible for screening and data extraction; however, we implemented quality assurance processes to address this concern. Two reviewers screened a 10% sample of the records, arriving at a 98% agreement. We also used second and third reviewers to support the critical appraisal and GRADE CERQual analysis of our results.

For the purposes of our review, we did not search Embase because we did not have institutional access. Guidance from the Cochrane Rapid Reviews Methods Group on the rapid review of randomized control trial data indicates that Embase should be searched if access is available.^68^ Noting the limitation in accessing this database, we followed best guidance and searched recommended specialized databases that were appropriate for the retrieval of qualitative studies, specifically CINAHL (EBSCO) and PsycINFO (Ovid).

Using the CFIR to guide data analysis also may have influenced the framing of our findings. Important findings may have been missed if they did not map onto existing domains and constructs in the CFIR. Several of the included studies recruited participants from ongoing randomized control trials on telemedicine; therefore, it is unclear whether these participants are a good reflection of those seen in clinical practice. Furthermore, our results are limited to those with MSK disorders treated by mental health professionals, occupational therapists, physicians, and physiotherapists; and therefore, may not be generalizable to the experiences of patients with other disorders treated by other healthcare professionals.

Finally, our review offers an overarching analysis of the lived experience of telehealth and does not offer specific considerations for how those experiences differ for people with varying MSK disorders. Future reviews, and indeed qualitative research, are needed to more substantively investigate the nuanced differences in how the experience of synchronous telehealth is lived by people with MSK disorders.

## Conclusion

Our findings, as refracted through the CFIR and rated with moderate confidence according to GRADE CERQual criteria, suggest that the experience of telehealth both before and during the pandemic was shaped by many factors including but not limited to patient perceptions of telehealth, existing relationships with practitioners, availability and accessibility of telehealth technologies, and perceptions about the importance of the role of the physical exam in assessing and treating MSK disorders. We have identified five clinical implications arising from our review which we believe hold importance for endeavoring to improve the patient experience of telehealth in the non-pharmacological management of MSK disorders.

## Appendix A

### Database: Ovid MEDLINE search strategy

1 Whiplash Injuries/

2 Neck Injuries/

3 Neck Pain/

4 Neck Muscles/in [Injuries]

5 exp Cervical Vertebrae/in [Injuries]

6 Radiculopathy/

7 exp Brachial Plexus Neuropathies/

8 exp Torticollis/

9 exp Back Injuries/

10 exp Back Pain/

11 Coccyx/in [Injuries]

12 Intervertebral Disc Displacement/

13 Intervertebral Disc Degeneration/

14 Lumbar Vertebrae/in [Injuries]

15 Lumbosacral Region/in [Injuries]

16 Osteoarthritis, Spine/

17 Piriformis Muscle Syndrome/

18 Sciatica/

19 Spinal Diseases/

20 Spinal Stenosis/

21 Thoracic Injuries/

22 Rib Fractures/

23 Intercostal Muscles/

24 Chest Pain/

25 Angina Pectoris/

26 Microvascular Angina/

27 Tietze's Syndrome/

28 Shoulder Pain/

29 exp Cumulative Trauma Disorders/

30 exp Median Neuropathy/

31 Shoulder Impingement Syndrome/

32 Shoulder Joint/in [Injuries]

33 Shoulder Injuries/

34 exp Arm Injuries/

35 exp Hand Injuries/

36 Wrist Injuries/

37 Finger Injuries/

38 Radial Neuropathy/

39 exp Ulnar Neuropathies/

40 Thoracic Outlet Syndrome/

41 exp Hip Injuries/

42 exp Knee Injuries/

43 exp Foot Injuries/

44 exp Toes/in [Injuries]

45 Ankle Injuries/

46 Lateral Ligament, Ankle/in [Injuries]

47 Fasciitis, Plantar/

48 tendinosis.ab,ti.

49 tendinopathy.ab,ti.

50 Musculoskeletal Pain/

51 exp Headache Disorders/

52 Headache/

53 Post-Traumatic Headache/

54 Tension-Type Headache/

55 (headache* adj4 (tension type or tension-type or tension or muscle contraction or psychomyogenic or stress or essential or ideopathic or psychogenic or daily-persistent)).ab,ti.

56 (headache* adj4 (whiplash or WAD)).ab,ti.

57 (headache* adj4 (post traumatic or post-traumatic or PTH)).ab,ti.

58 (headache* adj4 (cervicogenic or cervical or neck or pericranial)).ab,ti.

59 exp Temporomandibular Joint Disorders/

60 exp Temporomandibular Joint/

61 Jaw/in [Injuries]

62 Mastication/

63 whiplash*.mp.

64 (neck* adj3 (pain* or injur* or ach* or myalg* or symptom* or syndrome* or discomfort* or sore* or impairment* or disorder* or dysfunction* or tear* or imping* or sprain* or strain*)).mp.

65 neckach*.mp.

66 (brachial* adj2 (plexus adj2 neuropath*)).mp.

67 torticollis*.mp.

68 Polyradiculopathy/

69 (cervical* adj3 (pain* or injur* or ach* or myalg* or symptom* or syndrome* or discomfort* or sore* or impairment* or disorder* or dysfunction* or tear* or imping* or sprain* or strain*)).mp.

70 (cervicalg* or cervicodyn*).mp.

71 ((cervicobrach* or cervico-brach*) adj2 (pain* or injur* or ach* or myalg* or symptom* or syndrome* or discomfort* or sore* or impairment* or disorder* or dysfunction* or tear* or imping* or sprain* or strain*)).mp.

72 (brachial* adj2 (plexus adj2 (pain* or injur* or ach* or myalg* or symptom* or syndrome* or discomfort* or sore* or impairment* or disorder* or dysfunction* or tear* or imping* or sprain* or strain*))).mp.

73 ((cervicogen* or cervico-gen*) adj3 (pain* or injur* or ach* or myalg* or symptom* or syndrome* or discomfort* or sore* or impairment* or disorder* or dysfunction* or tear* or imping* or sprain* or strain*)).mp.

74 ((c-spine* or “c spine”) adj3 (pain* or injur* or ach* or myalg* or symptom* or syndrome* or discomfort* or sore* or impairment* or disorder* or dysfunction* or tear* or imping* or sprain* or strain*)).mp.

75 (radiculopath* or radiating* or radicular*).mp.

76 Brachial Plexus/in [Injuries]

77 Cervical Plexus/in [Injuries]

78 Low Back Pain/

79 (back adj3 (pain* or injur* or ach* or symptom* or myalg* or syndrome* or discomfort* or sore* or impairment* or disorder* or dysfunction* or tear* or imping* or sprain* or strain*)).mp.

80 (backach* or back-ach*).mp.

81 back-pain*.mp.

82 (low-back adj3 (pain* or injur* or ach* or symptom* or myalg* or syndrome* or discomfort* or sore* or impairment* or disorder* or dysfunction* or tear* or imping* or sprain* or strain*)).mp.

83 (lower-back adj3 (pain* or injur* or ach* or myalg* or symptom* or syndrome* or discomfort* or sore* or impairment* or disorder* or dysfunction* or tear* or imping* or sprain* or strain*)).mp.

84 ((disc or discs) adj3 (pain* or extru* or degenerat* or displac* or herniat* or prolaps* or sequestered or slipped or protru* or avuls*)).mp.

85 (disk* adj3 (pain* or extru* or degenerat* or displac* or herniat* or prolaps* or sequestered or slipped or protru* or avuls*)).mp.

86 (lumbar* adj3 (pain* or injur* or ach* or myalg* or symptom* or syndrome* or discomfort* or sore* or impairment* or disorder* or dysfunction* or tear* or imping* or sprain* or strain*)).mp.

87 (lumbo* adj3 (pain* or injur* or ach* or myalg* or symptom* or syndrome* or discomfort* or sore* or impairment* or disorder* or dysfunction* or tear* or imping* or sprain* or strain*)).mp.

88 (lower-trunk* adj3 (pain* or injur* or ach* or myalg* or symptom* or syndrome* or discomfort* or sore* or impairment* or disorder* or dysfunction* or tear* or imping* or sprain* or strain*)).mp.

89 (coccydyn* or coccygodyn* or coccalg* or coccygalg* or coccygeal*).mp.

90 (coccyx* adj3 (pain* or injur* or ach* or myalg* or symptom* or syndrome* or discomfort* or sore* or impairment* or disorder* or dysfunction* or tear* or imping* or sprain* or strain*)).mp.

91 (tailbone* adj3 (pain* or injur* or ach* or myalg* or symptom* or syndrome* or discomfort* or sore* or impairment* or disorder* or dysfunction* or tear* or imping* or sprain* or strain*)).mp.

92 (dorsalg* or lumbago*).mp.

93 lumboischialg*.mp.

94 (piriformis* adj3 (pain* or injur* or ach* or myalg* or symptom* or syndrome* or discomfort* or sore* or impairment* or disorder* or dysfunction* or tear* or imping* or sprain* or strain*)).mp.

95 (sacral* adj3 (pain* or injur* or ach* or myalg* or symptom* or syndrome* or discomfort* or sore* or impairment* or disorder* or dysfunction* or tear* or imping* or sprain* or strain*)).mp.

96 ((sacroiliac* or sacro-iliac*) adj3 (pain* or injur* or ach* or myalg* or symptom* or syndrome* or discomfort* or sore* or impairment* or disorder* or dysfunction* or tear* or imping* or sprain* or strain*)).mp.

97 ((sacrococcy* or sacro-coccy*) adj3 (pain* or injur* or ach* or myalg* or symptom* or syndrome* or discomfort* or sore* or impairment* or disorder* or dysfunction* or tear* or imping* or sprain* or strain*)).mp.

98 (SI adj2 (joint adj3 (pain* or injur* or ach* or myalg* or symptom* or syndrome* or discomfort* or sore* or impairment* or disorder* or dysfunction* or tear* or imping* or sprain* or strain*))).mp.

99 sciatic*.mp.

100 osteoarthrit*.mp.

101 spondyl*.mp.

102 (thoracolumbar* adj3 (pain* or injur* or ache* or myalg* or stenos* or discomfort* or dysfunction* or sore* or impairment* or syndrome* or disorder* or symptom* or tear* or imping* or sprain* or strain*)).mp.

103 (thoraco-lumbar* adj3 (pain* or injur* or ache* or myalg* or stenos* or discomfort* or dysfunction* or sore* or impairment* or syndrome* or disorder* or symptom* or tear* or imping* or sprain* or strain*)).mp.

104 (synovial* adj3 cyst*).mp.

105 (curvatur* adj3 (spine* or spinal*)).mp.

106 (vertebr* adj3 (pain* or injur* or ach* or myalg* or symptom* or syndrome* or discomfort* or sore* or impairment* or disorder* or dysfunction* or tear* or imping* or sprain* or strain*)).mp.

107 (discogen* adj3 (pain* or injur* or ach* or myalg* or symptom* or syndrome* or discomfort* or sore* or impairment* or disorder* or dysfunction* or tear* or imping* or sprain* or strain*)).mp.

108 (stenos?s* adj3 (spine* or spinal* or vertebr* or lumbar* or lumbao* or “low back” or low-back* or “lower back” or lower-back* or neck* or cervical*)).mp.

109 Intervertebral Disc/in [Injuries]

110 Sacrum/in [Injuries]

111 Ribs/in [Injuries]

112 Intercostal Muscles/in [Injuries]

113 Sternum/in [Injuries]

114 (thoracic* adj3 (pain* or injur* or ach* or myalg* or symptom* or syndrome* or discomfort* or sore* or impairment* or disorder* or dysfunction* or sprain* or strain*)).mp.

115 (thorax* adj3 (pain* or injur* or ach* or myalg* or symptom* or syndrome* or discomfort* or sore* or impairment* or disorder* or dysfunction* or sprain* or strain*)).mp.

116 (torso* adj3 (pain* or injur* or ach* or myalg* or symptom* or syndrome* or discomfort* or sore* or impairment* or disorder* or dysfunction* or sprain* or strain*)).mp.

117 ((sternum* or sternal*) adj3 (pain* or injur* or ach* or myalg* or symptom* or syndrome* or discomfort* or sore* or impairment* or disorder* or dysfunction* or sprain* or strain*)).mp.

118 ((sternoclavic* or sterno-clavic*) adj3 (pain* or injur* or ach* or myalg* or symptom* or syndrome* or discomfort* or sore* or impairment* or disorder* or dysfunction* or sprain* or strain*)).mp.

119 (pectoral* adj3 (pain* or injur* or ach* or myalg* or symptom* or syndrome* or discomfort* or sore* or impairment* or disorder* or dysfunction* or sprain* or strain*)).mp.

120 ((t-spine* or “t spine”) adj3 (pain* or injur* or ach* or myalg* or symptom* or syndrome* or discomfort* or sore* or impairment* or disorder* or dysfunction* or sprain* or strain*)).mp.

121 ((mid-back* or midback*) adj3 (pain* or injur* or ach* or myalg* or symptom* or syndrome* or discomfort* or sore* or impairment* or disorder* or dysfunction* or sprain* or strain*)).mp.

122 ((cervicothoracic* or cervico-thoracic*) adj3 (pain* or injur* or ach* or myalg* or symptom* or syndrome* or discomfort* or sore* or impairment* or disorder* or dysfunction* or sprain* or strain*)).mp.

123 (chest* adj2 wall* adj3 (pain* or injur* or ach* or myalg* or symptom* or syndrome* or discomfort* or sore* or impairment* or disorder* or dysfunction* or sprain* or strain*)).mp.

124 ((rib or ribs) adj3 (pain* or injur* or ach* or myalg* or symptom* or syndrome* or discomfort* or sore* or impairment* or disorder* or dysfunction* or sprain* or strain*)).mp.

125 ((costal* or intercostal* or inter-costal* or intracostal* or intra-costal*) adj3 (pain* or injur* or ach* or myalg* or symptom* or syndrome* or discomfort* or sore* or impairment* or disorder* or dysfunction* or sprain* or strain*)).mp.

126 ((cervicothoracic* or cervico-thoracic* or pector* or cervical* or pseudo*) adj3 angina*).mp.

127 ((costotransvers* or costochondral) adj3 (pain* or injur* or ach* or myalg* or symptom* or syndrome* or discomfort* or sore* or impairment* or disorder* or dysfunction* or sprain* or strain*)).mp.

128 ((costovertebr* or costo-vertebr*) adj3 (pain* or injur* or ach* or myalg* or symptom* or syndrome* or discomfort* or sore* or impairment* or disorder* or dysfunction* or sprain* or strain*)).mp.

129 ((costal adj3 chondrit*) or costochondrit*).mp.

130 (chest* adj2 wall* adj3 tender*).mp.

131 ((neck* or cervical*) adj3 tender*).mp.

132 (tietze* adj3 syndrome*).mp.

133 fibrositis*.mp.

134 (polyradicul* or poly-radicul*).mp.

135 ((slipping* or click* or displac* or syndrome*) adj3 (rib or ribs)).mp.

136 ((sternocostal* or sterno-costal*) adj3 (pain* or injur* or ach* or myalg* or symptom* or syndrome* or discomfort* or sore* or impairment* or disorder* or dysfunction* or sprain* or strain*)).mp.

137 (chest* adj3 (pain* or injur* or ach* or myalg* or symptom* or syndrome* or discomfort* or sore* or impairment* or disorder* or dysfunction* or sprain* or strain*)).mp.

138 Thoracic Vertebrae/in [Injuries]

139 Shoulder Injuries/

140 Rotator Cuff/

141 Thoracic Outlet Syndrome/

142 (thoracic* adj3 outlet*).mp.

143 (rotator* adj2 cuff* adj3 (pain* or injur* or ach* or myalg* or symptom* or syndrome* or discomfort* or sore* or impairment* or disorder* or dysfunction* or tear* or imping* or sprain* or strain*)).mp.

144 (shoulder* adj3 (pain* or injur* or ach* or myalg* or symptom* or syndrome* or discomfort* or sore* or impairment* or disorder* or dysfunction* or tear* or imping* or sprain* or strain*)).mp.

145 (painful* adj2 arc).mp.

146 (shoulder* adj3 (frozen* or capsul*)).mp.

147 (adhes* adj3 capsul*).mp.

148 ((glenohumeral* or scapul* or acromio* or subacrom* or supraspinatus* or infraspinatus* or subscapularis* or “teres minor” or “teres major” or trapezius* or deltoid*) adj3 (pain* or injur* or ach* or myalg* or symptom* or syndrome* or discomfort* or sore* or impairment* or disorder* or dysfunction* or tear* or imping* or sprain* or strain*)).mp.

149 Elbow/in [Injuries]

150 Elbow Tendinopathy/

151 Tennis Elbow/

152 Median Neuropathy/

153 Carpal Tunnel Syndrome/

154 (((upper* adj2 limb*) or upper-limb* or (upper* adj2 extremit*) or upper-extremit*) adj3 (pain* or injur* or ach* or myalg* or symptom* or syndrome* or discomfort* or sore* or impairment* or disorder* or dysfunction* or sprain* or strain*)).mp.

155 ((arm or arms) adj (pain* or injur* or ach* or myalg* or symptom* or syndrome* or discomfort* or sore* or impairment* or disorder* or dysfunction* or sprain* or strain*)).mp.

156 ((bicep* or bicipital* or coracobrachial*) adj3 (pain* or injur* or ach* or myalg* or symptom* or syndrome* or discomfort* or sore* or impairment* or disorder* or dysfunction* or sprain* or strain*)).mp.

157 (elbow* adj3 (pain* or injur* or ach* or myalg* or symptom* or syndrome* or discomfort* or sore* or impairment* or disorder* or dysfunction* or sprain* or strain*)).mp.

158 (forearm* adj3 (pain* or injur* or ach* or myalg* or symptom* or syndrome* or discomfort* or sore* or impairment* or disorder* or dysfunction* or sprain* or strain*)).mp.

159 epicondyl*.mp.

160 (tennis* adj3 elbow*).mp.

161 ((wrist or wrists) adj3 (pain* or injur* or ach* or myalg* or symptom* or syndrome* or discomfort* or sore* or impairment* or disorder* or dysfunction* or sprain* or strain*)).mp.

162 ((hand or hands) adj (pain* or injur* or ach* or myalg* or symptom* or syndrome* or discomfort* or sore* or impairment* or disorder* or dysfunction* or sprain* or strain*)).mp.

163 (hand-wrist* adj (pain* or injur* or ach* or myalg* or symptom* or syndrome* or discomfort* or sore* or impairment* or disorder* or dysfunction* or sprain* or strain*)).mp.

164 (carpal* adj3 (pain* or injur* or ach* or myalg* or symptom* or syndrome* or discomfort* or sore* or impairment* or disorder* or dysfunction* or sprain* or strain*)).mp.

165 (metacarpal* adj3 (pain* or injur* or ach* or myalg* or symptom* or syndrome* or discomfort* or sore* or impairment* or disorder* or dysfunction* or sprain* or strain*)).mp.

166 ((finger or fingers) adj3 (pain* or injur* or ach* or myalg* or symptom* or syndrome* or discomfort* or sore* or impairment* or disorder* or dysfunction* or trigger* or sprain* or strain*)).mp.

167 (neuropath* adj3 (ulnar* or lateral* or radial* or median*)).mp.

168 Leg Injuries/

169 (((lower* adj2 limb*) or lower-limb* or (lower* adj2 extremit*) or lower-extremit*) adj3 (pain* or injur* or ach* or myalg* or symptom* or syndrome* or discomfort* or sore* or impairment* or disorder* or dysfunction* or tear* or imping* or sprain* or strain*)).mp.

170 ((hip or hips) adj3 (pain* or injur* or ach* or myalg* or symptom* or syndrome* or discomfort* or sore* or impairment* or disorder* or dysfunction* or tear* or imping* or sprain* or strain*)).mp.

171 ((femoroacetabular* or (femoral* adj acetab*) or FAI) adj3 (pain* or injur* or ach* or myalg* or symptom* or syndrome* or discomfort* or sore* or impairment* or disorder* or dysfunction* or tear* or imping* or sprain* or strain*)).mp.

172 ((pelvic* or pelvis*) adj3 (pain* or injur* or ach* or myalg* or symptom* or syndrome* or discomfort* or sore* or impairment* or disorder* or dysfunction* or tear* or imping* or sprain* or strain*)).mp.

173 ((buttock* or gluteal* or gluteus*) adj3 (pain* or injur* or ach* or myalg* or symptom* or syndrome* or discomfort* or sore* or impairment* or disorder* or dysfunction* or tear* or imping* or sprain* or strain*)).mp.

174 (trochanter* adj3 (pain* or injur* or ach* or myalg* or symptom* or syndrome* or discomfort* or sore* or impairment* or disorder* or dysfunction* or tear* or imping* or sprain* or strain*)).mp.

175 ((leg or legs) adj3 (pain* or injur* or ach* or myalg* or symptom* or syndrome* or discomfort* or sore* or impairment* or disorder* or dysfunction* or tear* or imping* or sprain* or strain*)).mp.

176 ((thigh* or rectus or adductor* or vastus* or sartorius* or quadricep* or iliopsoas*) adj3 (pain* or injur* or ach* or myalg* or symptom* or syndrome* or discomfort* or sore* or impairment* or disorder* or dysfunction* or tear* or imping* or sprain* or strain*)).mp.

177 Buttocks/in [Injuries]

178 Thigh/in [Injuries]

179 Groin/in [Injuries]

180 Lower Extremity/in [Injuries]

181 Iliotibial Band Syndrome/

182 Quadriceps Muscle/in [Injuries]

183 (groin* adj3 (pain* or injur* or ach* or myalg* or symptom* or syndrome* or discomfort* or sore* or impairment* or disorder* or dysfunction* or tear* or imping* or sprain* or strain*)).mp.

184 Patellofemoral Joint/in [Injuries]

185 Patellofemoral Pain Syndrome/

186 Patella/in [Injuries]

187 Patellar Ligament/in [Injuries]

188 Anterior Cruciate Ligament Injuries/

189 Cartilage, Articular/in [Injuries]

190 Ligaments, Articular/in [Injuries]

191 Posterior Cruciate Ligament/in [Injuries]

192 exp Collateral Ligaments/in [Injuries]

193 Medial Collateral Ligament, Knee/in [Injuries]

194 Meniscus/in [Injuries]

195 (knee* adj3 (pain* or injur* or ach* or myalg* or symptom* or syndrome* or discomfort* or sore* or impairment* or disorder* or dysfunction* or tear* or imping* or sprain* or strain*)).mp.

196 (patell* adj3 (pain* or injur* or ach* or myalg* or symptom* or syndrome* or discomfort* or sore* or impairment* or disorder* or dysfunction* or tear* or imping* or sprain* or strain*)).mp.

197 (cruciat* adj3 (pain* or injur* or ach* or myalg* or symptom* or syndrome* or discomfort* or sore* or impairment* or disorder* or dysfunction* or tear* or imping* or sprain* or strain*)).mp.

198 ((menisc* adj3 (pain* or injur* or ach* or myalg* or symptom* or syndrome* or discomfort* or sore* or impairment* or disorder* or dysfunction* or tear* or imping* or sprain* or strain*)) not eye*).mp.

199 Osteoarthritis, Hip/

200 Osteoarthritis, Knee/

201 Tibial Meniscus Injuries/

202 Medial Tibial Stress Syndrome/

203 Menisci, Tibial/in [Injuries]

204 (iliotibial* adj3 (pain* or injur* or ach* or myalg* or symptom* or syndrome* or discomfort* or sore* or impairment* or disorder* or dysfunction* or tear* or imping* or sprain* or strain*)).mp.

205 ((hoffa* or “fat pad”) adj3 (pain* or injur* or ach* or myalg* or symptom* or syndrome* or discomfort* or sore* or impairment* or disorder* or dysfunction* or tear* or imping* or sprain* or strain*)).mp.

206 ((jumper* adj3 knee*) or Osgood-schlatter* or sinding-larsen-johansson*).mp.

207 (apophysit* or (athlet* adj3 pubalg*)).mp.

208 (hamstring* adj3 (pain* or injur* or ach* or myalg* or symptom* or syndrome* or discomfort* or sore* or impairment* or disorder* or dysfunction* or tear* or imping* or sprain* or strain*)).mp.

209 ((ischiofemoral* or ischio-femoral*) adj3 (pain* or injur* or ach* or myalg* or symptom* or syndrome* or discomfort* or sore* or impairment* or disorder* or dysfunction* or tear* or imping* or sprain* or strain*)).mp.

210 ((iliofemoral* or ilio-femoral*) adj3 (pain* or injur* or ach* or myalg* or symptom* or syndrome* or discomfort* or sore* or impairment* or disorder* or dysfunction* or tear* or imping* or sprain* or strain*)).mp.

211 ((iliopectineal* or ilio-pectineal*) adj3 (pain* or injur* or ach* or myalg* or symptom* or syndrome* or discomfort* or sore* or impairment* or disorder* or dysfunction* or tear* or imping* or sprain* or strain*)).mp.

212 ((iliotibial* or ilio-tibial*) adj3 (pain* or injur* or ach* or myalg* or symptom* or syndrome* or discomfort* or sore* or impairment* or disorder* or dysfunction* or tear* or imping* or sprain* or strain*)).mp.

213 (inguinal* adj3 (pain* or injur* or ach* or myalg* or symptom* or syndrome* or discomfort* or sore* or impairment* or disorder* or dysfunction* or tear* or imping* or sprain* or strain*)).mp.

214 (pectine* adj3 (pain* or injur* or ach* or myalg* or symptom* or syndrome* or discomfort* or sore* or impairment* or disorder* or dysfunction* or tear* or imping* or sprain* or strain*)).mp.

215 ((pubofemoral* or pubo-femoral*) adj3 (pain* or injur* or ach* or myalg* or symptom* or syndrome* or discomfort* or sore* or impairment* or disorder* or dysfunction* or tear* or imping* or sprain* or strain*)).mp.

216 ((tibialfemoral* or tibial-femoral*) adj3 (pain* or injur* or ach* or myalg* or symptom* or syndrome* or discomfort* or sore* or impairment* or disorder* or dysfunction* or tear* or imping* or sprain* or strain*)).mp.

217 (iliopsoas* adj3 (pain* or injur* or ach* or myalg* or symptom* or syndrome* or discomfort* or sore* or impairment* or disorder* or dysfunction* or tear* or imping* or sprain* or strain*)).mp.

218 (ankle* adj3 (pain* or injur* or ach* or myalg* or symptom* or syndrome* or discomfort* or sore* or impairment* or disorder* or dysfunction* or tear* or imping* or sprain* or strain*)).mp.

219 ((foot or feet*) adj3 (pain* or injur* or ach* or myalg* or symptom* or syndrome* or discomfort* or sore* or impairment* or disorder* or dysfunction* or tear* or imping* or sprain* or strain*)).mp.

220 (heel adj3 (pain* or injur* or ach* or myalg* or symptom* or syndrome* or discomfort* or sore* or impairment* or disorder* or dysfunction* or tear* or imping* or sprain* or strain*)).mp.

221 (plantar* adj3 (pain* or injur* or ach* or myalg* or symptom* or syndrome* or discomfort* or sore* or impairment* or disorder* or dysfunction* or tear* or imping* or sprain* or strain*)).mp.

222 (plantar* adj2 fasc*).mp.

223 ((talofibular* or calcaneofibular* or calcaneotibial* or tibio*) adj3 (pain* or injur* or ach* or myalg* or symptom* or syndrome* or discomfort* or sore* or impairment* or disorder* or dysfunction* or tear* or imping* or sprain* or strain*)).mp.

224 (tarsal* adj3 (pain* or injur* or ach* or myalg* or symptom* or syndrome* or discomfort* or sore* or impairment* or disorder* or dysfunction* or tear* or imping* or sprain* or strain*)).mp.

225 (metatarsal* adj3 (pain* or injur* or ach* or myalg* or symptom* or syndrome* or discomfort* or sore* or impairment* or disorder* or dysfunction* or tear* or imping* or sprain* or strain*)).mp.

226 (toe adj3 (pain* or injur* or ach* or myalg* or symptom* or syndrome* or discomfort* or sore* or impairment* or disorder* or dysfunction* or tear* or imping* or sprain* or strain*)).mp.

227 exp Jaw/in [Injuries]

228 ((temporomandibular* or temporo-mandibular* or (temporo adj mandibular*)) adj3 (pain* or injur* or ach* or myalg* or symptom* or syndrome* or discomfort* or sore* or impairment* or disorder* or dysfunction* or tear* or imping* or sprain* or strain*)).mp.

229 ((jaw* or mandib* or orofacial* or oro-facial* or hyoid* or “discus articularis” or (articular* adj disc) or (articular* adj disk)) adj3 (pain* or injur* or ach* or myalg* or symptom* or syndrome* or discomfort* or sore* or impairment* or disorder* or dysfunction* or tear* or imping* or sprain* or strain*)).mp.

230 (jaw* adj3 synov*).mp.

231 ((craniomandibular* or cranio-mandibular* or ((cranio adj mandibular*) or digastricus* or masseter* or pterygoid* or stylohyoideus* or temporalis*)) adj3 (pain* or injur* or ach* or myalg* or symptom* or syndrome* or discomfort* or sore* or impairment* or disorder* or dysfunction* or tear* or imping* or sprain* or strain*)).mp.

232 headach*.mp.

233 musculoskeletal*.mp.

234 (musculoskeletal* or musculo-skeletal*).mp.

235 (myofasc* adj3 (pain* or injur* or ach* or myalg* or symptom* or syndrome* or discomfort* or sore* or impairment* or disorder* or dysfunction* or tear* or imping* or sprain* or strain*)).mp.

236 "Sprains and Strains"/

237 exp Tendinopathy/

238 exp Cumulative Trauma Disorders/

239 Bursitis/

240 Musculoskeletal Diseases/

241 exp Musculoskeletal System/in [Injuries]

242 Spinal Injuries/

243 exp Muscle, Skeletal/in [Injuries]

244 Tendon Injuries/

245 Athletic Injuries/

246 ((spine* or spinal*) adj4 (condition* or degener* or diseas* or disabl* or disabilit* or disorder* or pain* or injur* or impairment* or instabilit* or symptom* or syndrome* or discomfort* or sore* or ache* or complaint*)).mp.

247 bursit*.mp.

248 (tendinopath* or tend?nos* or tend?nit*).mp.

249 (cumulative* adj3 trauma*).mp.

250 (repetit* adj3 (pain* or injur* or ach* or myalg* or symptom* or syndrome* or discomfort* or sore* or impairment* or disorder* or dysfunction* or tear* or imping* or strain* or sprain*)).mp.

251 Achilles Tendon/in [Injuries]

252 Myalgia/

253 (golf* adj3 elbow*).mp.

254 (achilles* adj3 (pain* or injur* or ach* or myalg* or symptom* or syndrome* or discomfort* or sore* or impairment* or disorder* or dysfunction* or tear* or imping* or sprain* or strain* or ruptur*)).mp.

255 exp Mononeuropathies/

256 exp Nerve Compression Syndromes/

257 (neuropath* adj3 (pain* or injur* or ach* or myalg* or symptom* or syndrome* or discomfort* or sore* or impairment* or disorder* or dysfunction* or imping*)).mp.

258 Sacroiliac Joint/in [Injuries]

259 exp tendon entrapment/ or de quervain disease/

260 Metatarsalgia/

251 **or/ 1-260**

252 Ethnology/

253 Ethnopsychology/

254 "Surveys and Questionnaires"/

255 Patient Participation/

256 exp qualitative research/

257 belief*.mp.

258 experience*.mp.

259 attitud*.mp.

260 perception*.mp.

261 motivation*.mp.

262 (narrat* not (narrative adj review*)).mp.

263 (action* adj2 (note* or research* or study or studies or work*)).mp.

264 perspectiv*.mp.

265 (opinion* adj3 patient*).mp.

266 (patient* adj3 (preferenc* or satisf*)).mp.

267 qualitativ*.mp.

268 (semi-structur* or semistructur* or unstructur*).mp.

269 ((content* or conversation* or discourse* or semantic*) adj3 analys*).mp.

270 (theme* or thematic*).mp.

271 (diary* or diaries*).mp.

272 ethno*.mp.

273 ((behavioral* or behavioural* or descriptive*) adj3 (note* or research* or study or studies or work* or analy*)).mp.

274 ((content* or discourse* or semantic*) adj3 (note* or research* or study or studies or work* or analy*)).mp.

275 (constant adj2 (comparative or comparison)).mp.

276 ((field* adj3 (note* or research* or study or studies or work* or analy*)) or fieldwork*).mp.

277 (focus adj group*).mp.

278 satisf*.mp.

279 (self-report* or selfreport* or “self report”).mp.

280 (framework* adj analy*).mp.

281 (grounded* adj theor*).mp.

282 interview*.mp.

283 (key adj informant*).mp.

284 (open adj end*).mp.

285 personal*.mp.

286 phenomenol*.mp.

287 (purposive* adj sampl*).mp.

288 (theoretical adj2 (sampl* or saturation)).mp.

289 (patient* adj3 (expectation* or journey*)).mp.

290 (mix* adj3 (method* or research* or study or studies or work* or analy*)).mp.

291 mixed-method*.mp.

292 ((participatory* or implementation* or naturalistic or feminist*) adj3 (research* or study or studies or work* or analy* or inquiry)).mp.

293 (observational adj3 analy*).mp.

294 (observation adj3 (participant* or non-participant*)).mp.

295 (case study or case studies or case file review or case management).mp.

296 (process adj2 evaluat*).mp.

297 (metasynthes* or meta-synthes*).mp.

298 (document* adj2 collect*).mp.

299 Attitude/

300 Diaries as Topic/

301 Focus Groups/

302 Grounded Theory/

303 Interviews as Topic/

304 Motivation/

305 Narration/

306 Patient Preference/

307 Patient Satisfaction/

308 Perception/

309 Personal Narrative/

310 Personal Narratives as Topic/

311 Personal Satisfaction/

312 Self Report/

313 exp Anthropology, Cultural/

314 exp Psychology/

315 exp Emotions/

316 exp Affect/

317 (survey* or questionnair*).mp.

318 exp Social Sciences/

319 (open-form or (open* adj2 form) or open-text* or (open* adj2 text*)).mp.

320 ((open adj end*) or open-end*).mp.

321 interview.pt.

322 (multimethod* or multi-method*).mp.

323 Behavioral Research/

324 **or/ 252-323**

325 Telemedicine/

326 Telerehabilitation/

327 Telenursing/

328 exp Telephone/

329 Remote Consultation/

330 Distance Counseling/

331 Videoconferencing/

332 Telecommunications/

333 Internet/

334 Internet-Based Intervention/

335 Smartphone/

336 (care adj3 (tele* or internet* or online* or remote* or virtual* or video* or digital* or distance* or long-distance* or distant* or electronic* or mobile)).mp.

337 (rehab* adj3 (tele* or internet* or online* or remote* or virtual* or video* or digital* or distance* or long-distance* or distant* or electronic* or home* or mobile)).mp.

338 (consult* adj3 (tele* or internet* or online* or remote* or virtual* or video* or digital* or distance* or long-distance* or distant* or electronic* or home* or mobile)).mp.

339 (collaborat* adj3 (tele* or internet* or online* or remote* or virtual* or video* or digital* or distance* or long-distance* or distant* or electronic* or home* or mobile)).mp.

340 (therap* adj3 (tele* or internet* or online* or remote* or virtual* or video* or digital* or distance* or long-distance* or distant* or electronic* or home* or mobile)).mp.

341 (treat* adj3 (tele* or internet* or online* or remote* or virtual* or video* or digital* or distance* or long-distance* or distant* or electronic* or home* or mobile)).mp.

342 (intervention* adj3 (tele* or internet* or online* or remote* or virtual* or video* or digital* or distance* or long-distance* or distant* or electronic* or home* or mobile)).mp.

343 (health adj4 (distance* or distant* or tele* or internet* or online* or remote* or virtual* or video* or digital* or long-distance* or mobile)).mp.

344 (coach* adj3 (tele* or internet* or online* or remote* or virtual* or video* or digital* or distance* or long-distance* or distant* or electronic* or home* or mobile)).mp.

345 (communicat* adj3 (tele* or internet* or online* or remote* or virtual* or video* or digital* or distance* or long-distance* or distant* or electronic* or home* or mobile)).mp.

346 (app adj2 (digital or distance or distant or electronic or home or mobile)).mp.

347 (phone or phones or cellphone* or smartphone* or iphone* or telephone*).mp.

348 (e-consult* or econsult* or e-health* or ehealth* or e-intervention* or e-treat* or e-therap*).mp.

349 (m-health* or mhealth* or web-based* or (web* adj2 based*) or cybermed* or cyber-med*).mp.

350 (telecare* or telecollaborat* or teleconsult* or teleconference* or teleeducat* or telehealth* or teleguid* or telediagnos* or telelearn* or telemed* or telementor* or telemetr* or telemonitor* or teleneurol* or telepediatric* or telepractice* or telepresence* or telerehab* or telerobotic* or telescreen* or teletransmi*).mp.

351 (tele care* or tele collaborat* or tele consult* or tele conference* or tele educat* or tele health* or tele guide* or tele diagnos* or tele learn* or tele med* or tele mentor* or tele monitor* or tele neurol* or tele pediatric* or tele practice or tele presence* or tele rehab* or tele robotic* or tele screen* or tele transmi*).mp.

352 (zoom or skype or facetime).mp.

353 ((distan* or remote* or video*) adj2 (consult* or deliver*)).mp.

354 (delivery* adj3 (tele* or internet* or online* or remote* or virtual* or video* or digital* or distance* or long-distance* or distant* or electronic* or home* or mobile)).mp.

355 (video conferenc* or videoconferenc*).mp.

356 (telenurs* or tele-nurs* or telephysio* or tele-physio*).mp.

357 ((teletherap* or tele-therap* or tele therap*) not (x-ray or radiat* or cobalt or gamma* or cesium)).mp.

358 **or/ 325-357**

359251 and 324 and 358

360 limit 359 to yr="2010 -Current”

361 (comment or clinical conference or clinical trial or congress or consensus development conference or controlled clinical trial or editorial or letter or meta analysis or observational study or randomized controlled trial or review or systematic review or guideline or practice guideline or validation study).pt.

362 360 not 361

363 exp Animals/

364 exp Humans/

365 363 not 364

366 362 not 365

367 limit 366 to english language

## Appendix B

### CFIR definitions—original and amended

In the place of organizations and stakeholders, we used the terminology *community of practitioners*, gesturing to the different health professionals who use or will use synchronous telehealth in the treatment of MSK disorders. Our analysis does not consider specific stakeholders or organizational structures but rather focuses on how patients describe their experiences with telehealth. We reconfigured the definition of culture to focus on the meanings, core values, beliefs, and assumptions imagined to be shared across the healthcare cultures of biomedicine, chiropractic, physical therapy, and rehabilitation sciences, which constitute the community of practitioners. Thus, our use of the term culture refers to the systems of knowledge production, modes of behavior, social norms, and roles and responsibilities that constitute and perpetuate the everyday practices of these fields.

**Table:**

Original CFIR definition			Definition amended for rapid review
**I. INTERVENTION CHARACTERISTICS** **The features of an intervention that might influence implementation**			
	**1.1 Relative Advantage**	Stakeholders’ perceptions of the advantage of implementing the intervention versus an alternative solution	Patient perception of the advantage of implementing non-pharmacological interventions delivered through synchronous telehealth as compared to in-person care
	**1.2 Adaptability**	The degree to which an intervention can be adapted, tailored, refined, or reinvented to meet local needs	The degree to which non-pharmacological interventions delivered through synchronous telehealth can be adapted, tailored, refined, or reinvented to meet patient needs
	**1.3 Cost**	Costs of the intervention and costs associated with implementing the intervention including investment, supply, and opportunity costs	Costs of non-pharmacological interventions delivered through synchronous telehealth and costs associated with implementing telehealth
**II. OUTER SETTING** **The features of the external context or environment that might influence the implementation of synchronous telehealth**			
	**2.1 Patient needs and resources**	The extent to which patient needs, as well as barriers and facilitators to meet those needs, are accurately known and prioritized by the organization	The extent to which patient needs specific to the use of synchronous telehealth in the non-pharmacological management of musculoskeletal disorders, as well as barriers and facilitators to meet those needs, are accurately known and prioritized by the broader community of practitioners
**III. INNER SETTING** **The features of the implementing community of practitioners that might influence implementation**			
	**3.1 Culture**	Norms, values, and basic assumptions of a given organization	Norms, values, and basic assumptions shaping how care for MSK disorders is expected, provided, and enacted.
**IV. CHARACTERISTICS OF INDIVIDUALS** **The characteristics of patients that might influence implementation**			
	**4.1 Knowledge and beliefs about the intervention**	Individual's attitudes toward and value placed on the interventions as well as familiarity with facts, truths, and principles related to the intervention	Patient's attitudes toward and value placed on receiving non-pharmacological interventions delivered through synchronous telehealth as well as familiarity with facts, truths, and principles related to telehealth
	**4.2 Self-efficacy**	Individual belief in their own capabilities to execute course of action to achieve implementation goals	Patient's belief in their own capabilities to participate in the experience of receiving non-pharmacological interventions delivered through synchronous telehealth
	**4.3 Other personal attributes**	A broad construct to include other personal traits such as tolerance of ambiguity, intellectual ability, motivation, values, competence, capacity, and learning style	A broad construct to include other personal experiences associated with receiving non-pharmacological interventions delivered through synchronous telehealth
